# Statistical finite elements for misspecified models

**DOI:** 10.1073/pnas.2015006118

**Published:** 2020-12-28

**Authors:** Connor Duffin, Edward Cripps, Thomas Stemler, Mark Girolami

**Affiliations:** ^a^Department of Mathematics and Statistics, The University of Western Australia, Perth, WA 6009, Australia;; ^b^Complex Systems Group, The University of Western Australia, Perth, WA 6009, Australia;; ^c^Department of Engineering, University of Cambridge, Cambridge CB2 1PZ, United Kingdom;; ^d^Lloyd’s Register Foundation Programme for Data-Centric Engineering, The Alan Turing Institute, London NW1 2DB, United Kingdom

**Keywords:** Bayesian calibration, finite element methods, model discrepancy

## Abstract

We present a statistical finite element method for nonlinear, time-dependent phenomena, illustrated in the context of nonlinear internal waves (solitons). We take a Bayesian approach and leverage the finite element method to cast the statistical problem as a nonlinear Gaussian state–space model, updating the solution, in receipt of data, in a filtering framework. The method is applicable to problems across science and engineering for which finite element methods are appropriate. The Korteweg–de Vries equation for solitons is presented because it reflects the necessary complexity while being suitably familiar and succinct for pedagogical purposes. We present two algorithms to implement this method, based on the extended and ensemble Kalman filters, and demonstrate effectiveness with a simulation study and a case study with experimental data. The generality of our approach is demonstrated in *SI Appendix*, where we present examples from additional nonlinear, time-dependent partial differential equations (Burgers equation, Kuramoto–Sivashinsky equation).

## Significance

Science and engineering have benefited greatly from the ability of finite element methods (FEMs) to simulate nonlinear, time-dependent complex systems. The recent advent of extensive data collection from such complex systems now raises the question of how to systematically incorporate these data into finite element models, consistently updating the solution in the face of mathematical model misspecification with physical reality. This article describes general and widely applicable methodology for the coherent synthesis of data with FEM models, providing a data-driven probability distribution that captures all sources of uncertainty in the pairing of FEM with measurements.

The central role of physically derived, nonlinear, time-dependent partial differential equations (PDEs) in scientific and engineering research is undisputed, as is the need for numerical intervention in order to understand their behavior. The finite element method (FEM) has emerged as the foremost strategy to undergo this numerical intervention, yet when these discretized solutions are compared with empirical evidence, elements of model mismatch are revealed that require statistical formalisms to be dealt with appropriately (1–3). To address this problem of model misspecification, in this paper we introduce stochastic forcing inside the PDE and update the FEM discretized PDE solution with data in a filtering context.

Stochastic forcing is introduced through a random function within the governing equations. This represents an unknown process, which may have been omitted in the formulation of the physical model. For an elliptic linear PDE with coefficients Λ, this can be expressed as

$$\left\{\begin{array}{c} L_{\Lambda }u=f+\xi_{\theta },\;\xi_{\theta }∼GP\left(0,C_{\theta }\right),\;\\ u≔u\left(x\right),\;f≔f\left(x\right),\;x\in \Omega \subset R^{d}.\; \end{array}\right.$$

The push forward of the Gaussian random field $\xi_{\theta }$, with covariance parameters θ, induces a probability measure over the space of admissible solutions to the above. To embed this into a finite element model, we start with the weak form

$$A_{\Lambda }\left(u,v\right)=\left\langle f,v\right\rangle ,+\left\langle \xi_{\theta },v\right\rangle ,$$

where $A_{\Lambda }\left(\cdot ,\cdot \right)$ is the bilinear form generated from $L_{\Lambda }$ and ⟨⋅,⋅⟩ is the appropriate Hilbert space inner product. Discretizing with finite elements $u\left(x\right)=\sum_{i=1}^{M}u_{i}\phi_{i}\left(x\right)$, $v\left(x\right)=\sum_{i=1}^{M}v_{i}\varphi_{i}\left(x\right)$ yields the Gaussian measure over the solution FEM coefficients $u=\left(u_{1},u_{2},\ldots ,u_{M}\right)\in R^{M}$:

$$p\left(u\;|\;\Lambda ,\theta \right)=N\left(A^{-1}b,A^{-1}G\left(\theta \right)A^{-T}\right),$$

where $A_{ij}=A_{\Lambda }\left(\phi_{i},\varphi_{j}\right)$, $b_{i}=\left\langle f,\varphi_{i}\right\rangle$, and $G{\left(\theta \right)}_{ij}=\left\langle \phi_{i},C_{\theta }\varphi_{j}\right\rangle$. This defines a (finite-dimensional) prior distribution over the FEM model, which represents all assumed knowledge before observing data. The mean is the standard Galerkin solution, and the covariance results from the action of the discretized PDE operator on the covariance G(θ); further details are contained in *SI Appendix*, section 1. This was first developed in ref. 4, and we demonstrate the generality of such an approach by extending it to nonlinear, time-dependent PDEs.

An area in which nonlinear and time-dependent problems are ubiquitous is ocean dynamic processes, where essentially all problems stem from a governing system of nonlinear, time-dependent equations (e.g., the Navier–Stokes equations). The ocean dynamics community has grown increasingly cognizant of the importance of accurate uncertainty quantification (5, 6), with many possible applications [e.g., rogue waves (7), turbulent flow (8)] for our proposed methodology.

An example process is nonlinear internal waves (solitons), which are observed as waves of depression or elevation along a pycnocline in a density-stratified fluid and are of broad interest to both the scientific and engineering communities (9–13). The classical mathematical model for solitons is the Korteweg–de Vries (KdV) equation (14):

$$\left\{\begin{array}{c} u_{t}+\alpha uu_{x}+\beta u_{xxx}+cu_{x}=0,\;\\ u≔u\left(x,t\right),x\in \left[0,L\right],t\in \left[0,T\right],\; \end{array}\right.$$ [1]

where u is the pycnocline displacement. Coefficients α, β, and c are determined by physical parameters. Eq. **1** is readily interpretable: waves propagate at wave speed c, nonlinear steepening results from $uu_{x}$, and dispersion is due to $u_{xxx}$. Relative coefficient values determine the dominating regime, and waves can vary from quasilinear to highly nonlinear.

Despite KdV being well studied (15) and widely applied (16–18), its relative simplicity makes it prone to model mismatch. To compensate for this mismatch, we update the FEM discretized solution with observations in a filtering context. The resulting statistical FEM (statFEM) is shown using simulated and experimental data to

*i*) Approximate the data-generating process with a statistically coherent uncertainty quantification.
*ii*) Synthesize physics and data to give an interpretable posterior distribution.
*iii*) Utilize sparsely observed data to reconstruct observed phenomena.
*iv*) Enable the application of simpler physical models, updated with observations.

For practitioners faced with data, we believe these benefits are of importance, and we demonstrate the generality of our method in *SI Appendix* with further examples. Code to replicate the analysis is freely available online.*

## A Nonlinear, Time-Evolving Statistical FEM

A Gaussian process (GP), $\xi_{\theta }$, is introduced inside of the governing equations, which represent an unknown forcing process in space and time, with time-varying parameters θ. For a general nonlinear PDE, this is given by

$$u_{t}+L_{\Lambda }u+F_{\Lambda }\left(u\right)+\xi_{\theta }=0,\;\xi_{\theta }∼GP\left(0,C_{\theta }\right),$$ [2]

where $L_{\Lambda }$ and $F_{\Lambda }$ represent linear and nonlinear differential operators, respectively, with coefficients Λ. The push forward of the Gaussian measure $\xi_{\theta }$ induces a probability measure over the space of admissible solutions to Eq. **2** and characterizes our prior belief in the model based on modeling assumptions. The kernel of the covariance operator $C_{\theta }$ is given by

$$E\left[\xi_{\theta }\left(x,t\right)\xi_{\theta }\left(x^{\prime },t^{\prime }\right)\right]=k_{\theta }\left(x,x^{\prime }\right)\cdot \delta \left(t,t^{\prime }\right).$$

The exact form of this covariance can be decided upon by domain experts so that the uncertainty induced is physically motivated. For example, $k_{\theta }$ can be chosen to be a Matérn covariance function to reflect the unknown forcing having derivatives up to a known order. We assume a white noise process in time to facilitate the application of standard Kalman methods to solve the filtering problem; this is also convention in stochastic differential equations (19). When computing the prior defined by Eq. **2**, we use fixed parameters θ. When conditioning on data, we take an empirical Bayes approach and estimate θ through the log-marginal posterior.

Coefficients of Eq. **2** are assumed to be known, and we choose to update the numerical solution to the model, acknowledging that estimating Λ [using, e.g., maximum likelihood methods (20), Markov chain Monte Carlo (21), or inversion methods in general (22)] is also of utmost interest.

Discretizing Eq. **2** using finite elements in space with an implicit or explicit Euler method in time,^†^ denote by $u^{n}\in R^{M}$ the FEM coefficients at time nΔt, for time step Δt. Further analysis of this discretization will be the focus of future work. For a potentially nonlinear system of equations $F_{\Lambda }$, the resultant system can be expressed as (full construction is given in *SI Appendix*, sections 2 and 3)

$$F_{\Lambda }\left(u^{n},u^{n-1}\right)+e_{n-1}=0.$$

The vector $e_{n-1}$ represents Galerkin discretized increments of a Brownian motion process, $e_{n-1}∼N\left(0,\Delta_{t}G\left(\theta_{n}\right)\right)$.

Unlike the elliptic example in the Introduction, for Eq. **2** the induced probability measure on the FEM coefficients is not available in closed form, and we present two approximations, based on the extended Kalman filter (EKF) and the ensemble Kalman filter (EnKF). The first linearizes about the current solution with the Jacobian J of the nonlinear $F_{\Lambda }$ (evaluated at the current solution) to give a Gaussian approximation of $p\left(u^{n}\;|\;\theta_{1:n},\Lambda \right)\approx N\left(m_{n},C_{n}\right)$. The second uses an ensemble in which a perturbed system is solved, with realizations from $e_{n}$, at each time step. Summary statistics (e.g., mean, covariance) are then computed from this ensemble.

For the prior, the deterministic FEM solution is identically equal to the mean in the EKF approach. However we have found in numerical experiments that the EKF method, due to the use of the Jacobian, inflates the covariance at points of high gradient with reduction at points approaching near-zero gradient, when using large time steps. This does not occur with the EnKF approach.

**Table:** 

Algorithm 1: EKF algorithm
for $n\leq n_{t}$ do
(Prediction step)
Solve $F\left(m_{n|n-1},m_{n-1|n-1}\right)=0$.
${\hat{C}}_{n|n-1}={\left(J^{n}\right)}^{-1}\left(J^{n-1}C_{n-1|n-1}{\left(J^{n-1}\right)}^{⊤}\right){\left.mbJ^{n}\right)}^{-⊤}$
Estimate:
${\text{arg max}}_{\theta_{n},\sigma_{n}}\left\{\log p\left(y_{n}\;|\;y_{1:n-1},\theta_{1:n},\sigma_{1:n}\right)\right.$
$\left.+\log p\left(\theta_{n}\right)+\log p\left(\sigma_{n}\right)\right\}$.
$C_{n|n-1}={\hat{C}}_{n|n-1}+\Delta_{t}{\left(J^{n}\right)}^{-1}G\left(\theta_{n}\right){\left.J^{n}\right)}^{-⊤}$.
$S_{n}=H_{n}C_{n|n-1}H_{n}^{⊤}+\sigma_{n}^{2}I$.
(Analysis step)
$m_{n|n}=m_{n|n-1}+C_{n|n-1}H_{n}^{⊤}S_{n}^{-1}\left(y_{n}-H_{n}m_{n|n-1}\right)$.
$C_{n|n}=C_{n|n-1}-C_{n|n-1}H_{n}^{⊤}S_{n}^{-1}H_{n}C_{n|n-1}$.


## Conditioning on Data.

Data $y_{n}\in R^{N}$ are observed at time nΔt on the grid $x_{\text{obs}}$. These data are corrupted with noise $\eta_{n}∼N\left(0,\sigma_{n}^{2}I\right)$ independent to the model $u^{n}$ to give the data-generating process $y_{n}=H_{n}u^{n}+\eta_{n}$, where the linear observation operator $H_{n}:R^{M}\to R^{N}$ maps from the computed solution grid to the observation grid, using the FEM interpolant.

The filtered distribution $p\left(u^{n}\;|\;y_{1:n},\theta_{1:n},\sigma_{1:n},\Lambda \right)$, where $y_{1:n}=\left(y_{1},y_{2},\ldots ,y_{n}\right)$, is our primary object of interest.^‡^ We take a Bayesian interpretation and refer to this as the filtered posterior distribution or just the posterior, when the context is clear. However, as this is a filtering problem, non-Bayesian methods are perfectly valid. We assume that all distributions are Gaussian, so the posterior can be computed with standard methods in data assimilation (23); we use the EKF (24) and the EnKF (stochastic form) (25).

The initial conditions are known (i.e., they are given a Dirac measure). For time n, we make a tentative prediction step according to the PDE model $F_{\Lambda }$, propagating uncertainty in the previous time step [described by $p\left(u^{n-1}\;|\;y_{1:n-1},\theta_{1:n-1},\sigma_{1:n-1}\right)$], to give the prediction measure $p\left(u^{n}\;|\;y_{1:n-1},\theta_{1:n-1},\sigma_{1:n-1}\right)$. Parameters $\left(\theta_{n},\sigma_{n}\right)$ are then estimated, and the full prediction step is completed to estimate $p\left(u^{n}\;|\;y_{1:n-1},\theta_{1:n},\sigma_{1:n}\right)$. Data observed at time $n\Delta_{t}$ are then conditioned on to give the updated filtering distribution $p\left(u^{n}\;|\;y_{1:n},\theta_{1:n},\sigma_{1:n}\right)$.

We assume the parameters are independent across time [i.e., $p\left(\theta_{n}\;|\;\theta_{n-1}\right)=p\left(\theta_{n}\right)$]. Parameters may also be time constant, which is discussed in the maximum likelihood setting in ref. 26 and in the hierarchical Bayesian setting in ref. 27. *SI Appendix* also contains a possible modification of the method that accounts for time-invariant parameters. The following procedure provides an overview of the method, and *Algorithms 1* and *2* give pseudocode versions.

### Conditioning procedure.

At time n, assume that the measure on the previous time is described by

$$p\left(u^{n-1}\;|\;y_{1:n-1},\theta_{1:n-1},\sigma_{1:n-1}\right)=N\left(m_{n-1|n-1},C_{n-1|n-1}\right).$$

Then, proceed as follows:

*i*) Compute the tentative prediction step:

$$\begin{array}{ll} & p\left(u^{n}\;|\;y_{1:n-1},\theta_{1:n-1},\sigma_{1:n-1}\right)\\ & \;=\int p\left(u^{n}\;|\;u^{n-1}\right)p\left(du^{n-1}\;|\;y_{1:n-1},\theta_{1:n-1},\sigma_{1:n-1}\right)\\ & \;\approx N\left({\hat{m}}_{n|n-1},{\hat{C}}_{n|n-1}\right). \end{array}$$

*i*) Maximize the EKF log-marginal posterior to estimate parameters:

$$\begin{array}{ll} & {\text{arg max}}_{\theta_{n},\sigma_{n}}\left\{\log p\left(y_{n}\;|\;y_{1:n-1},\theta_{1:n},\sigma_{1:n}\right)\right.\\ & \;\left.+\log p\left(\theta_{n}\right)+\log p\left(\sigma_{n}\right)\right\}, \end{array}$$

*i*) where $\hat{G}\left(\theta_{n}\right)=\Delta_{t}{\left(J^{n}\right)}^{-1}G\left(\theta_{n}\right){\left(J^{n}\right)}^{-⊤}$,

$$\begin{array}{ll} & p\left(y_{n}\;|\;y_{1:n-1},\theta_{1:n},\sigma_{1:n}\right)\\ & \;=N\left(H_{n}{\hat{m}}_{n|n-1},H_{n}{\hat{C}}_{n|n-1}H_{n}^{⊤}+H_{n}\hat{G}\left(\theta_{n}\right)H_{n}^{⊤}+\sigma_{n}^{2}I\right). \end{array}$$

*i*) Compute the full prediction step:

$$\begin{array}{ll} & p\left(u^{n}\;|\;y_{1:n-1},\theta_{1:n},\sigma_{1:n-1}\right)\\ & \;=\int p\left(u^{n}\;|\;u^{n-1},\theta_{n}\right)p\left(du^{n-1}\;|\;y_{1:n-1},\theta_{1:n-1},\sigma_{1:n-1}\right)\\ & \;\approx N\left(m_{n|n-1},C_{n|n-1}\right). \end{array}$$

*i*) Complete the analysis step:

$$\begin{array}{ll} & p\left(u^{n}\;|\;y_{1:n},\theta_{1:n},\sigma_{1:n}\right)\\ & \;\propto p\left(y_{n}\;|\;u^{n},\sigma_{n}\right)p\left(u^{n}\;|\;y_{1:n-1},\theta_{1:n},\sigma_{1:n-1}\right)\\ & \;=N\left(m_{n|n},C_{n|n}\right). \end{array}$$

The prior $p\left(u^{n}\;|\;\theta_{n},\Lambda \right)$ is recovered if only the full prediction step (step 3) is completed at each iteration; completing the full sequence gives the posterior. Optimization of the log-marginal posterior is done using the limited-memory Broyden–Fletcher–Goldfarb–Shanno algorithm (28) with starting points set to the previous estimates. This log-marginal posterior is calculable due to the Gaussian assumption made in step 1. Prior information on hyperparameters is incorporated through $p\left(\theta_{n}\right)$ and $p\left(\sigma_{n}\right)$, which regularizes the optimization problem.

### Simulation Study.

We condition on data generated from an extended Korteweg–de Vries (eKdV) equation with a cubic nonlinear term:

$$w_{t}+\alpha ww_{x}+\beta w_{xxx}+\varepsilon w^{3}w_{x}=0,$$

**Table:** 

Algorithm 2: EnKF algorithm
for $n\leq n_{t}$ do
(Prediction step)
for $i\leq N_{ens}$ do
Solve $F\left(u_{pred}^{n,\left[i\right]},u^{n-1,\left[i\right]}\right)=0$.
Compute $m_{n|n-1}$ and ${\hat{C}}_{n|n-1}$ from $\left\{u_{pred}^{n,\left[i\right]}\right\}$
Estimate:
${\text{arg max}}_{\theta_{n},\sigma_{n}}\left\{\log p\left(y_{n}\;|\;y_{1:n-1},\theta_{1:n},\sigma_{1:n}\right)\right.$
$\left.+\log p\left(\theta_{n}\right)+\log p\left(\sigma_{n}\right)\right\}$
for $i\leq N_{ens}$ do
Solve $F\left(u_{pred}^{n,\left[i\right]},u^{n-1,\left[i\right]}\right)+e_{n-1}^{\left[i\right]}=0$.
Compute $m_{n|n-1}$ and ${\hat{C}}_{n|n-1}$ from $\left\{u_{pred}^{n,\left[i\right]}\right\}$
$S_{n}=H_{n}C_{n|n-1}H_{n}^{⊤}+\sigma_{n}^{2}I$.
(Analysis step)
for $i\leq N_{ens}$ do
$u^{n,\left[i\right]}=u_{pred}^{n,\left[i\right]}+C_{n|n-1}H_{n}^{⊤}S_{n}^{-1}\left(y_{n}+\eta_{n}^{\left[i\right]}-H_{n}u_{pred}^{n,\left[i\right]}\right)$


setting α=1, β=0.01, and ε=20. The misspecified KdV model

$$u_{t}+\alpha uu_{x}+\beta u_{xxx}+\xi_{\theta }=0$$ [3]

has the same coefficient values, with initial conditions set to a wave of depression $u\left(x,0\right)=-0.3{\text{sech}}^{2}\left(x-15\right)$ on the space–time grid (x,t)∈[0,20]×[0,50]. Boundary conditions are periodic. Gaussian random forcing $\xi_{\theta }$ has spatial covariance $k_{\theta }\left(x,x^{\prime }\right)=\tau^{2}\exp\left(-∥x-x^{\prime }{∥}^{2}/\left(2\ell^{2}\right)\right)$ (we refer to τ as the scale parameter and ℓ as the length parameter).

KdV is discretized following ref. 29, using $P_{1}$ trial functions and $P_{0}$ testing functions, with a Crank–Nicolson method in time. The data-generating process is simulated using Dedalus (30) with 1,024 grid points in space. This is then down sampled to 20 grid points and jittered with synthetic Gaussian observational noise (mean 0, variance $0.001^{2}$) to give the simulated dataset.

**Fig. 1.**
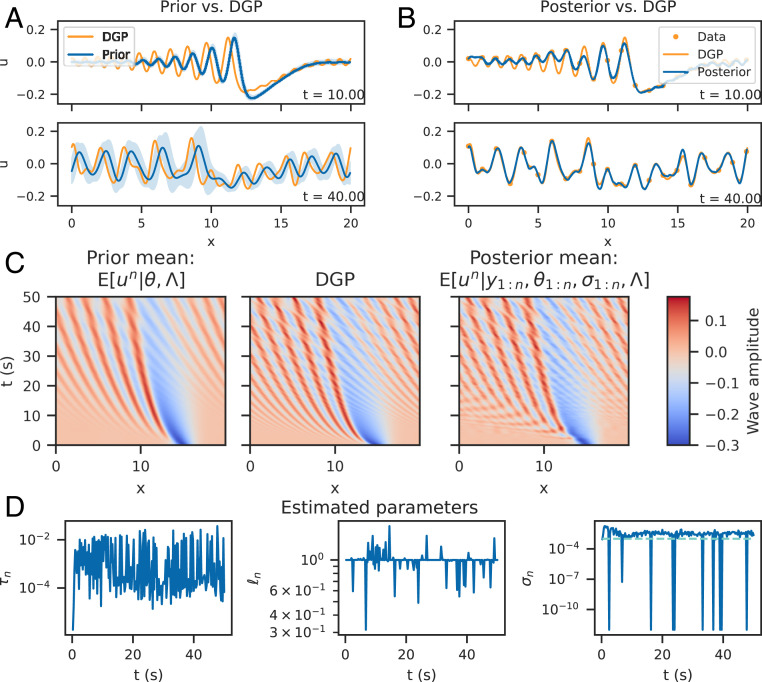
Simulation study results (using EnKF). *A* shows a prior solution with fixed hyperparameters $\theta_{n}$ with the data-generating process (DGP; as labeled) for two times with accompanying 95% credible intervals. Note the degree of model mismatch between the two profiles. *B* shows the DGP and the posterior mean and 95% credible intervals for the same times as the prior. Given the data, the model is highly certain about the updated mean, for which the model mismatch has been corrected. This is now using estimated hyperparameters $\theta_{n}$ for a fully data-driven approach. *C* shows the prior mean, DGP, and posterior mean over the entire simulation grid in space and time, and *D* shows the estimated parameters $\theta_{n}$, $\sigma_{n}$ across time, with the true value for $\sigma_{n}$ shown as a dashed turquoise line.

We assume $y_{n}=Hu^{n}+\eta_{n}$, where $u^{n}$ is the Galerkin discretized solution to Eq. **3** and $\eta_{n}∼N\left(0,\sigma_{n}^{2}I\right)$. Hyperparameters $\theta_{n}=\left(\tau_{n},\ell_{n}\right)$ and noise level $\sigma_{n}$ are estimated at each step by maximizing the log-marginal posterior, with the weakly informative truncated Gaussian priors $\tau_{n}∼N_{+}\left(1,1^{2}\right)$, $\ell_{n}∼N_{+}\left(1,1^{2}\right)$, and $\sigma_{n}∼N_{+}\left(0,1^{2}\right)$. The EnKF is used in this section, with $n_{x}=400$, $n_{t}=2,001$, and $N_{ens}=400$. Results are presented in Fig. 1.

For a fixed set of hyperparameters $\tau_{n}=0.0025$, $\ell_{n}=1$ for all n, the data-generating process and estimated prior are shown in Fig. 1*A*, appearing visually mismatched in the mean via phase shift, increased oscillations, and increased wave interactions. Note that the stochastic forcing induces an uncertainty about the PDE solution, represented by the 95% credible intervals shown. Note also that the data-generating process is approximately contained within the credible intervals.

Fig. 1*B* shows that the posterior mean approximates the data-generating process, and the posterior uncertainty bounds have shrunk as a result of conditioning, indicating high certainty about the posterior mean values. Model discrepancy between the data and the statFEM solution has been corrected for. The space–time view of the posterior, shown in Fig. 1*C*, shows that the posterior has incorporated the complex soliton interactions in the data, not present in the prior.

Parameter estimates (Fig. 1*D*) indicate that the length and noise parameters are both stable, with the noise being slightly overestimated (i.e., $\sigma_{n}\approx 0.003>0.001$). Times at which the noise is not identified result in it being set to the lower bound. The scale parameter quantifies the accuracy of the model prediction step at each time step. In this case, model predictions vary in their accuracy and appear approximately bounded to within $\left(10^{-5},10^{-1}\right)$.

## Case Study: Experimental Data

We now apply the method to the experimental data collected in ref. 31. Experiments were conducted to study weakly nonlinear models for internal waves in lakes and consisted of generating internal waves in a two-layer stratified system, inside of a clear acrylic tank of dimensions 6×0.3×0.29 m. The tank contained an upper layer of fresh water and a lower layer of saline water, with a density gradient of $\Delta \rho =20\;{\text{kgm}}^{-3}$. The tank was able to rotate in order to establish the initial conditions, which were an inclined plane of angle 𝜗=0.5°. This initial condition mimics the shear induced by strong winds in lakes. At time t=0, the tank is rotated to restore it to the horizontal.

**Fig. 2.**
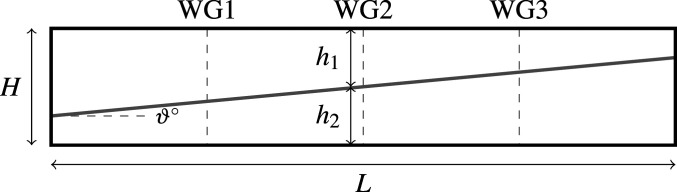
Schematic diagram of the experimental apparatus. Wave gauges (WGs) are labeled WG1, WG2, and WG3, and the initial conditions are shown as a gray line, labeled with initial angle 𝜗°.

**Fig. 3.**
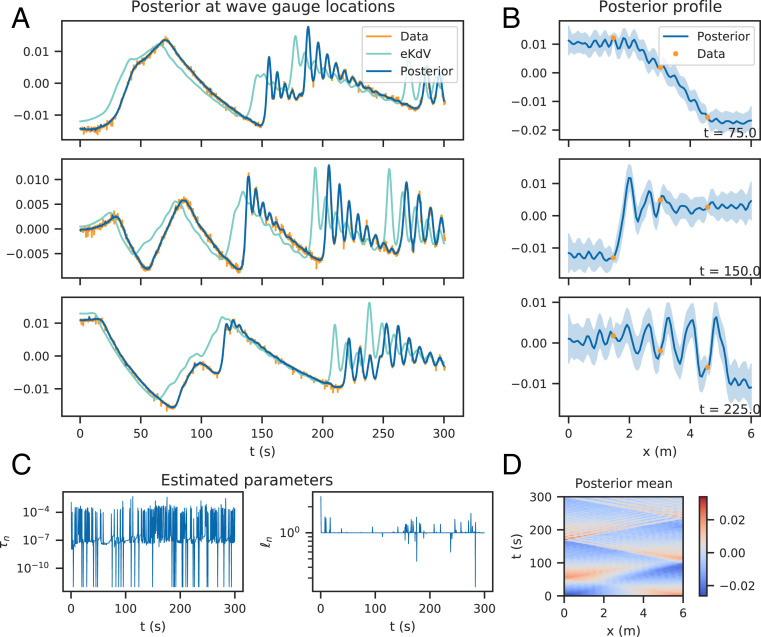
Experimental data results; posterior computed by the EnKF. *A* shows the observed data, the deterministic solution to the misspecified model, and the posterior mean at the wave gauges with 95% credible intervals up to time T=300 s. Model mismatch between the model and data is visually apparent and has been offset in the posterior mean. *B* shows the posterior mean wave profile inside of the tank, with 95% credible intervals and the data, for three times. The wave profile has been reconstructed by conditioning on the observed data. *C* shows the estimated hyperparameters $\theta_{n}$, and *D* shows the estimated posterior mean over the space–time grid.

Data were recorded at three spatially equidistant locations in the tank using ultrasonic wave gauges (Fig. 2), taking measurements approximately every 0.01 s, up to T=1,000 s; we use data up to T=300 s. Data are measured in voltages and are postprocessed to give pynocline displacements in meters. These data are plotted in Fig. 3, where the small measurement error is visually apparent. Transient behavior is observed before steepening, and a soliton wave train forms; three such steepening events are observed in the data we analyze. As T→1,000 s, dissipation results in the wave profile approaching a flat steady-state profile.

Our physical model is an eKdV equation with—for computational simplicity—a linear dissipation term. We acknowledge that for laminar boundaries, other methods are preferred (31, 32). Including some form of dissipation is important as otherwise, the model becomes impractically mismatched by the end of the simulation. The eKdV is given by

$$u_{t}+\alpha uu_{x}+\beta u_{xxx}+cu_{x}+\nu u+\xi_{\theta }=0$$ [4]

for u≔u(x,t), x∈[0,L], t∈[0,T], and with coefficients

$$\alpha =\frac{3}{2}\frac{c\left(h_{1}-h_{2}\right)}{h_{1}h_{2}},\;\beta =\frac{ch_{1}h_{2}}{6},\;c=\sqrt{\frac{g^{\prime }h_{1}h_{2}}{H}},\;g^{\prime }=\frac{\Delta \rho g}{\rho_{0}}.$$

We set $\xi_{\theta }$ as a GP as described previously, with spatial covariance kernel $k_{\theta }$ set to a squared exponential with scale and length hyperparameters τ and ℓ. For the experiment under consideration, we have $h_{1}=0.232\;\text{m}$, $h_{2}=0.058\;\text{m}$, $H=h_{1}+h_{2}=0.29\;\text{m}$, and $\rho_{0}=1,000\;{\text{kgm}}^{-3}$. The dissipation coefficient ν is an inverse timescale, which is set to $3\times 10^{-3}\;{\text{s}}^{-1}$.

Incorporation of reflective boundary conditions is done by solving the eKdV equation across the extended domain [0,2L] with periodic boundary conditions and summing solutions in the (reflected) subdomains [0,L], [L,2L]:

$$u_{\text{exact}}\left(x,t\right)≔u\left(x,t\right)+u\left(2L-x,t\right),\;x\in \left[0,L\right].$$

Details on the derivation are in ref. 31. Solutions to the deterministic version of Eq. **4** at the locations of the wave gauges are shown in Fig. 3. We show the deterministic solution instead of the prior due to accumulation of errors for large simulation times.

The eKdV model does not capture the observed behavior exactly. The model waves have higher velocity than the observations, and model amplitudes are slightly larger than observed amplitudes. It is conjectured that this is due to misparameterization of dissipation, but in any case, the model is misspecified. Rather than estimating the eKdV parameters using inversion techniques, we sequentially update the model with observations to give the posterior $p\left(u^{n}\;|\;y_{1:n},\theta_{1:n},\sigma_{1:n}\right)$.

As before, we assume $y_{n}=Hu^{n}+\eta_{n}$ with known noise $\eta_{n}∼N\left(0,1.3588\times 10^{-8}I\right)$. As we solve on the extended domain [0,2L] and sum solutions, the observation operator H is taken to be the sum of the appropriate function values given by our FEM interpolant (a linear operation). The observation points are unchanging, as is the solution mesh, so H is constant in time. The hyperparameters of $\xi_{\theta }$, $\theta_{n}=\left(\tau_{n},\ell_{n}\right)$, must be estimated at each iteration by maximizing the log-marginal posterior. Due to small data in space (three observations each time step), we use a projection method to estimate hyperparameters. This linearly projects the predicted mean $m_{n|n-1}$ forward, estimated from the data points: $y_{i}^{n}=a_{n}+b_{n}m_{n|n-1}\left(x_{i}\right)$. Parameters $a_{n},b_{n}$ are estimated to give the best least squares linear projection from the prediction to the data. This gives a projected dataset, ${\tilde{y}}_{n}$, using the linear shift: ${\tilde{y}}_{n}=a_{n}+b_{n}m_{n|n-1}$. The estimated hyperparameters are then given by ${\text{arg max}}_{\theta_{n}}\left\{\log p\left({\tilde{y}}_{n}\;|\;y_{1:n-1},\theta_{1:n},\sigma_{1:n}\right)+\log p\left(\theta_{n}\right)\right\}$, in which the observed data $y_{n}$ are replaced with the projected data ${\tilde{y}}_{n}$. We project to a grid of 100 points uniformly spaced across the solution grid. Note that this is only for the parameter estimation step, and we do not use this ${\tilde{y}}_{n}$ as the data in the analysis step.

We set weakly informative priors: $\tau_{n}∼N_{+}\left(1,1^{2}\right)$ and $\ell_{n}∼N_{+}\left(1,1^{2}\right)$. The posterior is computed using the ensemble method with $n_{x}=200$, $n_{t}=1,001$, and $N_{ens}=2,048$. Results are shown in Fig. 3. The posterior mean values of $p\left(u^{n}\;|\;y_{1:n},\theta_{1:n},\sigma_{1:n}\right)$ at the wave gauges are shown in Fig. 3*A* and offer a close fit to the data in comparison with the eKdV solution. The credible intervals shrink about the data (compare Fig. 3*B*) and are not seen on the figure.

Posterior wave profiles are shown in Fig. 3*B* and demonstrate that given the data, the method is able to yield a sensible estimate for the underlying wave profile and is hence able to reconstruct the wave profile given sparse observations in space (Fig. 3*D*). Furthermore the provided uncertainty quantification is physically sensible, with bounds contracting about the data and expanding near the boundaries.

The hyperparameters, $\theta_{n}$, are shown in Fig. 3*C*. The scale parameter is seen to vary between two distinct levels, indicating that the model predictions vary in their accuracy. The amplitude of these mismatch scales shows that in this case, model mismatch is a cumulative effect that takes some time before it is obviously occurring (Fig. 3*A*, eKdV solution). Repeated conditioning on data helps to mitigate these long timescale effects due to continual updating. A space–time view of the posterior mean wave profile is shown in Fig. 3*D*, demonstrating that the general behavior of the flow (e.g., reflective boundary conditions, dissipation, wave train formation) is indeed captured.

## Conclusions

We present a data-driven approach to the FEM that assimilates observations into nonlinear, time-dependent PDEs by embedding model misspecification uncertainty in the governing equations and sequentially updating the discretized equations with observations in a filtering context. Examples presented using the KdV equation (and the additional systems studied in *SI Appendix*) demonstrate that the method can approximate the data-generating process to give an interpretable posterior distribution, which reconstructs the observed phenomena.

This work sets the foundation for future studies of embedding data within FEM models. The use of the underlying Kalman framework permits the drawing upon of ideas from high-dimensional data assimilation, which for the systems studied here, were not needed. Techniques from Bayesian inversion can also be used to provide uncertainty quantification for physical quantities of interest, which will also allow for more accurate prediction. Finally, we believe that the development of similar methodology for alternate discretizations (e.g., spectral methods) could also be of great benefit, allowing for even broader application.

## Supplementary Material

Supplementary File

## Data Availability

Data and code have been deposited on GitHub (https://github.com/connor-duffin/statkdv-paper).

## Footnotes

*It is available at https://github.com/connor-duffin/statkdv-paper.

^†^Crank–Nicolson may also be used to ensure stability.

^#x2021;^From here on in, we implicitly condition on PDE coefficients Λ.

## References

[1] Kennedy M. C., O’Hagan A., Bayesian calibration of computer models. J. Roy. Stat. Soc. B 63, 425–464 (2001).

[2] Judd K., Smith L. A., Indistinguishable states II: The imperfect model scenario. Phys. Nonlinear Phenom. 196, 224–242 (2004).

[3] Berger J. O., Smith L. A., On the statistical formalism of uncertainty quantification. Annu Rev Stat Appl. 6, 433–460 (2019).

[4] Girolami M., Febrianto E., Yin G., Cirak F., The statistical finite element method (statFEM) for coherent synthesis of observation data and model predictions. Comput. Methods Appl. Mech. Eng., 10.17863/CAM.59639 (2020).

[5] Lermusiaux P. F. J., Uncertainty estimation and prediction for interdisciplinary ocean dynamics. J. Comput. Phys. 217, 176–199 (2006).

[6] Fringer O. B., Dawson C. N., He R., Ralston D. K., Zhang Y. J., The future of coastal and estuarine modeling: Findings from a workshop. Ocean Model. 143, 101458 (2019).

[7] Alam M. R., Predictability horizon of oceanic rogue waves. Geophys. Res. Lett. 41, 8477–8485 (2014).

[8] Majda A. J., Branicki M., Lessons in uncertainty quantification for turbulent dynamical systems. Discrete Contin. Dyn. Syst. Ser. A 32, 3133–3221 (2012).

[9] Osborne A. R., Burch T. L., Internal solitons in the Andaman Sea. Science 208, 451–460 (1980). 17744535 10.1126/science.208.4443.451

[10] Boegman L., Stastna M., Sediment resuspension and transport by internal solitary waves. Annu. Rev. Fluid Mech. 51, 129–154 (2019).

[11] Cacchione D., Pratson L. F., Ogston A., The shaping of continental slopes by internal tides. Science 296, 724–727 (2002). 11976451 10.1126/science.1069803

[12] Wang Y. H., Dai C. F., Chen Y. Y., Physical and ecological processes of internal waves on an isolated reef ecosystem in the South China Sea. Geophys. Res. Lett. 34, L18609 (2007).

[13] Huang X., An extreme internal solitary wave event observed in the northern South China Sea. Sci. Rep. 6, 30041 (2016). 27444063 10.1038/srep30041 PMC4956752

[14] Korteweg D. J., de Vries G., On the change of form of long waves advancing in a rectangular canal, and on a new type of long stationary waves. Lond. Edinb. Dubl. Phil. Mag. J. Sci. 39, 422–443 (1895).

[15] Drazin P. G., Johnson R. S., Solitons: An Introduction (Cambridge University Press, 1989).

[16] Lamb K. G., Yan L., The evolution of internal wave undular bores: Comparisons of a fully nonlinear numerical model with weakly nonlinear theory. J. Phys. Oceanogr. 26, 2712–2734 (1996).

[17] Holloway P. E., Pelinovsky E., Talipova T., A generalized Korteweg-de Vries model of internal tide transformation in the coastal zone. J. Geophys. Res. Oceans 104, 18333–18350 (1999).

[18] Helfrich K. R., Melville W. K., Long nonlinear internal waves. Annu. Rev. Fluid Mech. 38, 395–425 (2006).

[19] Øksendal B., Stochastic Differential Equations (Springer, 2003).

[20] Ionides E. L., Bretó C., King A. A., Inference for nonlinear dynamical systems. Proc. Natl. Acad. Sci. U.S.A. 103, 18438–18443 (2006). 17121996 10.1073/pnas.0603181103 PMC3020138

[21] Girolami M., Bayesian inference for differential equations. Theor. Comput. Sci. 408, 4–16 (2008).

[22] Stuart A. M., Inverse problems: A Bayesian perspective. Acta Numer. 19, 451–559 (2010).

[23] Law K., Stuart A., Zygalakis K., Data Assimilation (Springer, Cham, Switzerland, 2015).

[24] Jazwinski A. H., Stochastic Processes and Filtering Theory (Courier Corporation, 2007).

[25] Evensen G., The ensemble Kalman filter: Theoretical formulation and practical implementation. Ocean Dynam. 53, 343–367 (2003).

[26] Shumway R. H., Stoffer D. S., Time Series Analysis and Its Applications: With R Examples(Springer Texts in Statistics, Springer International Publishing, ed. 4, 2017).

[27] Katzfuss M., Stroud J. R., Wikle C. K., Ensemble Kalman methods for high-dimensional hierarchical dynamic space-time models. J. Am. Stat. Assoc., 1–43 (2019).

[28] Nocedal J., Wright S., Numerical Optimization (Springer Science & Business Media, 2006).

[29] Debussche A., Printems J., Numerical simulation of the stochastic Korteweg–de Vries equation. Phys. Nonlinear Phenom. 134, 200–226 (1999).

[30] Burns K. J., Vasil G. M., Oishi J. S., Lecoanet D., Brown B. P., Dedalus: A flexible framework for numerical simulations with spectral methods. Phys. Rev. Res. 2, 023068 (2020).

[31] Horn D., Imberger J., Ivey G., Redekopp L., A weakly nonlinear model of long internal waves in closed basins. J. Fluid Mech. 467, 269–287 (2002).

[32] Grimshaw R., Pelinovsky E., Talipova T., Damping of large-amplitude solitary waves. Wave Motion 37, 351–364 (2003).

