# Supplementary material for: Statistical finite elements for misspecified models
Source: Proc Natl Acad Sci U S A. 2020 Dec 28;118(2):e2015006118. doi: 10.1073/pnas.2015006118 (PMC7812808; doi:10.1073/pnas.2015006118)
Supplement: Supplementary File [file pnas.2015006118.sapp.pdf]

PNAS
www.pnas.org

Supplementary Information for

Statistical finite elements for misspecified models

Connor Duffin, Edward Cripps, Thomas Stemler and Mark Girolami

Corresponding author: Connor Duffin
E-mail: connor.duffin@research.uwa.edu.au

This PDF file includes:

Figs. S1 to S4
References for SI reference citations

1. Linear, static statFEM

An elliptic PDE with coefficients Λ can be written as:

$$\begin{cases} L_\Lambda u = f + \xi_\theta, \text{ in } \Omega, \quad \xi_\theta \sim \mathcal{GP}(0, k_\theta(\cdot,\cdot)), \\ u = 0 \text{ on } \partial\Omega, \\ u := u(x), \; f := f(x), \; x \in \Omega \subset \mathbb{R}^d. \end{cases} \qquad [1]$$

We will now derive a Gaussian measure μ_0 which serves as the prior reference measure. Typical analysis of the deterministic problem begins by looking for weak solutions to Eq. (1) by multiplying by testing functions $\psi \in H_0^1(\Omega)$ assuming that $f \in L^2(\Omega)$. The space $H_0^1(\Omega)$ denotes the Sobolev space with first-order weak derivatives in $L^2(\Omega)$ that vanish on $\partial\Omega$.

To start we show that if we assume $\xi_\theta \in L^2(\Omega)$ then we can define a Gaussian measure on $L^2(\Omega)$. Writing the L^2 inner product as $\langle f, g\rangle = \int_\Omega f(x)g(x)\,\mathrm{d}x$, which is understood as a Lebesgue integral, we can define a Gaussian measure $\mathcal{N}(0, C_\theta)$ from the random field as the covariance operator C_θ is defined from the kernel k_θ (see (1), Chapter 6), i.e.

$$(C_\theta h)(x) = \int k_\theta(x, x') h(x')\,\mathrm{d}x',$$

so $\xi_\theta \sim \mathcal{N}(0, C_\theta)$ on $L^2(\Omega)$.

We now return to Eq. (1) and multiply by test functions $\psi \in H_0^1(\Omega)$ — also assuming solutions $u \in H_0^1(\Omega)$ — and integrating over the problem domain Ω to give the weak form:

$$\mathcal{A}_\Lambda(u, \psi) = \langle f, \psi\rangle, + \langle \xi_\theta, \psi\rangle$$

where $\mathcal{A}_\Lambda(\cdot,\cdot)$ is the bilinear form generated from L_Λ. We note that it is assumed $f, \xi_\theta \in L^2(\Omega)$. The Sobolev space $H_0^1(\Omega)$ has an orthonormal basis $\{\phi_i\}_{i\in\mathbb{N}}$ so $u = \sum_{i=1}^\infty u_i \phi_i(x)$ and thus

$$\sum_{i=1}^{\infty} u_i \mathcal{A}_\Lambda(\phi_i, \psi) = \langle f, \psi\rangle, + \langle \xi_\theta, \psi\rangle.$$

Now as $\psi \in H_0^1(\Omega)$ also we can write without loss of generality

$$\sum_{i=1}^{\infty} u_i \mathcal{A}_\Lambda(\phi_i, \phi_j) = \langle f, \phi_j\rangle, + \langle \xi_\theta, \phi_j\rangle, \quad j \in \mathbb{N}.$$

The above can be viewed as an infinite system of equations with the matrix A having entries $A_{ij} = \mathcal{A}_\Lambda(\phi_i, \phi_j)$, and thus can be viewed as an operator on ℓ^2 as $Au = \left\{\sum_{i=1}^\infty u_i \mathcal{A}_\Lambda(\phi_i, \phi_j)\right\}_{j\in\mathbb{N}}$. We assume the ℓ^2 structure for the following theorem.

Theorem 1. *The operator $A : \ell^2 \to \ell^2$ is invertible.*

Proof. If A is not invertible then $Au = 0$ for some $u \neq 0$. We show that if $Au = 0$ and $u \neq 0$ that this leads to a contradiction.

$$\begin{aligned} \langle Au, u\rangle &= \sum_i u_i \sum_j \mathcal{A}_\Lambda(\phi_i, \phi_j) u_j \\ &= \sum_i \sum_j \mathcal{A}_\Lambda(u_i\phi_i, u_j\phi_j) \\ &= \mathcal{A}_\Lambda\left(\sum_i u_i\phi_i, \sum_j u_j\phi_j\right) \\ &= \mathcal{A}_\Lambda(u, u) \geq |C| \|u\|^2 > 0, \end{aligned}$$

where the last inequality is because $u \neq 0$ and is established from the coercivity of the bilinear form (2). This contradicts $Au = 0$. ☐

Then for $b = \{\langle f, \phi_j\rangle\}_j$, $\xi = \{\langle \xi_\theta, \phi_j\rangle\}_j$, we have $u = A^{-1}(b + \xi)$. Thus $\mathbb{E}u = A^{-1}b$, and

$$\mathbb{E}\left[(u - \mathbb{E}u) \otimes (u - \mathbb{E}u)\right] = A^{-1}GA^{-1}, \quad G_{ij} = \int \phi_i(x) \int k_\theta(x, x')\phi_j(x')\,\mathrm{d}x'\mathrm{d}x.$$

So $\{u_i\}_i \sim \mu_0 = \mathcal{N}(A^{-1}b, A^{-1}GA^{-1})$ and so we write $u \sim \mu_0 = \mathcal{N}(m_u, C_u)$ (the dominating measure for the posterior).

Using the Radon-Nikodym derivative we can then define the posterior measure μ^y on function space (1) under the condition that the posterior is absolutely continuous with respect to the prior. This gives the (infinite-dimensional) posterior (Z a normalizing constant):

$$\frac{\mathrm{d}\mu^y}{\mathrm{d}\mu_0} = \frac{1}{Z}\exp\left(-\frac{1}{2}\|\mathbf{y} - Hu\|^2_{\sigma^2\mathbf{I}}\right).$$

With $H : H_0^1(\Omega) \to \mathbb{R}^N$. In this work we assume that H is a linear operator that maps from the function space to the data space and typically refer to it as the observation operator. The data-generating-process is assumed to have Gaussian measurement error given by $\mathcal{N}(0, \sigma^2\mathbf{I})$. Note the posterior measure as written above implicitly conditions on PDE parameters Λ, and covariance parameters θ.

In order to permit computation, however, we need to project from the infinite dimensional Sobolev space to a finite dimensional subset U_h and do the same for the testing functions (i.e. projecting to $V_h \subset H_0^1(\Omega)$). One can take the projection operator $P_M u = \sum_{i=1}^M u_i \phi_i(x)$ and project to a finite-dimensional subset of a basis $\{\phi_i\}_i$ and thus as in the introduction we discretize: $u_h(x) = \sum_{i=1}^M u_{h,i}\phi_i(x)$, $v_h(x) = \sum_{i=1}^M v_{h,i}\psi_i(x)$, and

$$\mathcal{A}_\Lambda(u_h, \psi_j) = \langle f, \psi_j \rangle, + \langle \xi_\theta, \psi_j \rangle.$$

This then defines the (now finite-dimensional) Gaussian measure over the vector of FEM coefficients $\mathbf{u} = (u_{h,1}, u_{h,2}, \ldots)^\top$:

$$p(\mathbf{u} \mid \Lambda, \theta) = \mathcal{N}(\mathbf{A}^{-1}\mathbf{b}, \mathbf{A}^{-1}\mathbf{G}(\theta)\mathbf{A}^{-\top}),$$

where $\mathbf{A}_{ij} = \mathcal{A}_\Lambda(\phi_i, \psi_j)$, $\mathbf{b}_i = \langle f, \psi_i \rangle$, and $\mathbf{G}(\theta)_{ij} = \langle \phi_i, C_\theta \psi_j \rangle$. We write this as shorthand $p(\mathbf{u} \mid \theta, \Lambda) = \mathcal{N}(\mathbf{m}_u, \mathbf{C}_u)$.

The full covariance matrix $\mathbf{G}(\theta)$ can be written as

$$\mathbf{G}(\theta)_{ij} = \int_\Omega \psi_i(x) \int_\Omega k_\theta(x, x')\psi_j(x')\, \mathrm{d}x'\, \mathrm{d}x.$$

This covariance kernel can be chosen to encode information about the spatial variation of the process. For example, assuming that forcing is smooth in space means the popular squared exponential covariance kernel may be appropriate (we have used this covariance in all of our examples). There is a vast literature on covariance kernels; see (3), Chapter 4, for a thorough treatment.

Arrival of data $\mathbf{y}$ with some measurement error process η can be written as

$$\mathbf{y} = \mathbf{H}\mathbf{u} + \boldsymbol{\eta}, \quad \boldsymbol{\eta} \sim \mathcal{N}(\mathbf{0}, \sigma^2\mathbf{I}),$$

where $\mathbf{H} : \mathbb{R}^M \to \mathbb{R}^N$ is the now finite-dimensional linear observation operator. The finite-dimensional Bayes theorem gives the posterior distribution over the FEM coefficients

$$\begin{aligned} p(\mathbf{u} \mid \mathbf{y}, \theta, \sigma, \Lambda) &\propto p(\mathbf{y} \mid \mathbf{u}, \sigma) p(\mathbf{u} \mid \theta, \Lambda) \\ &= \mathcal{N}(\mathbf{m}, \mathbf{C}), \end{aligned}$$

in which

$$\mathbf{m} = \mathbf{m}_u + \mathbf{C}_u\mathbf{H}^\top \left(\mathbf{H}\mathbf{C}_u\mathbf{H}^\top + \sigma^2\mathbf{I}\right)^{-1} (\mathbf{y} - \mathbf{H}\mathbf{m}_u),$$

$$\mathbf{C} = \mathbf{C}_u - \mathbf{C}_u\mathbf{H}^\top \left(\mathbf{H}\mathbf{C}_u\mathbf{H}^\top + \sigma^2\mathbf{I}\right)^{-1} \mathbf{H}\mathbf{C}_u.$$

We note that throughout this paper that this should really be also conditional on the differential operators and forcing functions that form the dynamics, but this conditioning is taken as implicit so as to avoid cumbersome notation.

Parameters θ are estimated from the log-marginal likelihood $p(\mathbf{y} \mid \theta, \sigma, \Lambda) = \mathcal{N}\left(\mathbf{H}\mathbf{m}_u, \mathbf{H}\mathbf{C}_u\mathbf{H}^\top + \sigma^2\mathbf{I}\right)$ using either sampling or optimization based approaches depending on the need for uncertainty quantification.

2. Linear, time-dependent statFEM

Now we consider the parabolic time-dependent problem:

$$\begin{cases} \partial_t u + L_\Lambda u = f + \xi_\theta, & \xi_\theta \sim \mathcal{GP}(0, k_\theta(x, x') \cdot \delta(t - t')), \\ u := u(x),\ f := f(x),\ x \in \Omega \subset \mathbb{R}^d, & t \in [0, T]. \end{cases}$$

For mathematical simplicity we take the separable covariance function

$$\mathbb{E}\left[\xi_\theta(x, t)\xi_\theta(x', t')\right] = k_\theta(x, x') \cdot \delta(t, t'). \quad [2]$$

Which has the implication that stochastic forcing is white noise in time and spatially regular as per $k_\theta(\cdot, \cdot)$. We start by making a spatial discretization of the above with finite elements to give the semidiscrete problem and then use finite difference methods to give the fully discrete problem (see e.g. (4)). We start with the very similar weak form (with $u \in U$, $\psi \in V$):

$$\langle u_t, \psi \rangle + \mathcal{A}_\Lambda(u, \psi) = \langle f, \psi \rangle, + \langle \xi_\theta, \psi \rangle,$$

and project into finite dimensional subsets $U_h \subset U$, $V_h \subset V$, using finite elements $u_h(x, t) = \sum_{i \in I} u_{h,i}(t)\phi_i(x)$, $v_h(x, t) = \sum_{i \in I} v_{h,i}(t)\psi_i(x)$, to give the system of ODEs:

$$\langle \partial_t u_h, \psi_j \rangle + \mathcal{A}_\Lambda(u_h, \psi_j) = \langle f, \psi_j \rangle, + \langle \xi_\theta, \psi_j \rangle, \quad j \in I.$$

The covariance of $\langle \xi_\theta, \psi_j \rangle$ is defined by:

$$\mathbb{E}[\langle \xi_\theta, \psi_i \rangle \langle \xi_\theta, \psi_j \rangle] = \delta(t, t') \int_\Omega \psi_i(x) \int_\Omega k(x, x') \psi_j(x') \, \mathrm{d}x \, \mathrm{d}x'.$$

Which implies that the Gaussian process $\boldsymbol{\xi}_t = (\langle \xi, \psi_1 \rangle, \langle \xi, \psi_2 \rangle, \ldots)^\top$ can be described by:

$$\begin{aligned} \mathbb{E}[\boldsymbol{\xi}_t] &= \mathbf{0}, \\ \mathbb{E}[\boldsymbol{\xi}_t \boldsymbol{\xi}_{t'}] &= \delta(t, t') \cdot \mathbf{G}(\theta), \\ \mathbf{G}(\theta)_{ij} &= \int_\Omega \psi_i(x) \int_\Omega k_\theta(x, x') \psi_j(x') \, \mathrm{d}x' \, \mathrm{d}x. \end{aligned}$$

This $\boldsymbol{\xi}_t$ can be informally thought of as the derivative of a Brownian motion process $\boldsymbol{\beta}_t$ with diffusion matrix $\mathbf{G}$ (5).

We can concatenate the FEM coefficients into a vector $\mathbf{u} = (u_1(t), u_2(t), \ldots)^\top$ to write the above as a vector SDE:

$$\mathbf{M} \, \mathrm{d}\mathbf{u} + \mathbf{A}\mathbf{u} \, \mathrm{d}t = \mathbf{b} \, \mathrm{d}t + \mathrm{d}\boldsymbol{\beta}_t,$$

where $\mathbf{M}_{ij} = \langle \phi_i, \psi_j \rangle$ (the mass matrix) and $\mathbf{A}$ and $\mathbf{b}$ are as above. Next making a time discretization, $\mathbf{u}^n = (u_1(n\Delta_t), u_2(n\Delta_t), \ldots)^\top$, and using an explicit Euler discretization, one can write:

$$\mathbf{M}\left(\mathbf{u}^n - \mathbf{u}^{n-1}\right) + \Delta_t \mathbf{A}\mathbf{u}^{n-1} = \Delta_t \mathbf{b} + \mathbf{e_{n-1}},$$

in which $\mathbf{e}_{n-1} = \boldsymbol{\beta}_n - \boldsymbol{\beta}_{n-1} \sim \mathcal{N}(0, \Delta_t \mathbf{G}(\theta_n))$ are i.i.d. Gaussian (timesteps are assumed equal through the simulation). The implicit Euler follows

$$\mathbf{M}\left(\mathbf{u}^n - \mathbf{u}^{n-1}\right) + \Delta_t \mathbf{A}\mathbf{u}^{n} = \Delta_t \mathbf{b} + \mathbf{e_{n-1}},$$

so too the Crank-Nicolson

$$\mathbf{M}\left(\mathbf{u}^n - \mathbf{u}^{n-1}\right) + \Delta_t \mathbf{A}\mathbf{u}^{n+1/2} = \Delta_t \mathbf{b} + \mathbf{e_{n-1}},$$

where $\mathbf{u}^{n+1/2} := (\mathbf{u}^{n+1} + \mathbf{u}^n)/2$.

For exposition let us look at the the explicit Euler method for which the updating equation can be written as

$$\mathbf{u}^n = (\mathbf{I} - \Delta_t \mathbf{M}^{-1}\mathbf{A})\mathbf{u}^{n-1} + \Delta_t \mathbf{M}^{-1}\mathbf{b} + \mathbf{M}^{-1}\mathbf{e_{n-1}},$$

which defines the conditional measure at time n to be

$$\mathbf{u}^n \mid \mathbf{u^{n-1}}, \theta_n, \Lambda \sim \mathcal{N}\left(\left(\mathbf{I} - \Delta_t \mathbf{M}^{-1}\mathbf{A}\right)\mathbf{u}^{n-1} + \Delta_t \mathbf{M}^{-1}\mathbf{b}, \Delta_t \mathbf{M}^{-1}\mathbf{G}(\theta_n)\mathbf{M}^{-\top}\right).$$

The data are arriving at each timestep in the form $\mathbf{y}_n = \mathbf{H}_n \mathbf{u}^n + \boldsymbol{\eta}_n$ and have some Gaussian additive noise $\boldsymbol{\eta}_n \sim \mathcal{N}(\mathbf{0}, \sigma_n^2 \mathbf{I})$. This is a high-dimensional linear Gaussian state space problem. This class of models have been well studied (6) and one can apply standard Kalman filtering methods to obtain the filtering distribution $p(\mathbf{u}^n \mid \mathbf{y}_{1:n}, \theta_{1:n}, \sigma_{1:n}, \Lambda)$. We denote by $\mathbf{m}_{n|n-1}$ the posterior mean at time n conditional on data up to and including time $n-1$. The covariance also follows this notation. Under the assumption of unknown parameters θ, and a known previous filtering distribution, $\mathbf{u}^{n-1} \mid \mathbf{y}_{1:n-1}, \theta_{1:n-1}, \sigma_{1:n-1}, \Lambda \sim \mathcal{N}(\mathbf{m}_{n|n-1}, \mathbf{C}_{n|n-1})$, the filtering proceeds as:

1. Predict:

$$\mathbf{m}_{n|n-1} = (\mathbf{I} - \Delta_t \mathbf{M}^{-1}\mathbf{A})\mathbf{m}_{n-1|n-1} + \Delta_t \mathbf{M}^{-1}\mathbf{b},$$

$$\hat{\mathbf{C}}_{n|n-1} = (\mathbf{I} - \Delta_t \mathbf{M}^{-1}\mathbf{A})\mathbf{C}_{n-1|n-1}(\mathbf{I} - \Delta_t \mathbf{M}^{-1}\mathbf{A})^\top.$$

2. Estimate θ_n, σ_n:

$$\arg\max_{\theta_n, \sigma_n} \{\log p(\mathbf{y}_n \mid \mathbf{y}_{1:n-1}, \theta_{1:n}, \sigma_{1:n}) + \log p(\theta_n) + \log p(\sigma_n)\}$$

$$p(\mathbf{y}_n \mid \mathbf{y}_{1:n-1}, \theta_{1:n}, \sigma_{1:n}) = \mathcal{N}\left(\mathbf{H}_n \mathbf{m}_{n|n-1}, \mathbf{H}_n \hat{\mathbf{C}}_{n|n-1}\mathbf{H}_n^\top + \mathbf{H}_n \hat{\mathbf{G}}(\theta_n)\mathbf{H}_n^\top + \sigma_n^2 \mathbf{I}\right),$$

   where $\hat{\mathbf{G}}(\theta_n) = \Delta_t \mathbf{M}^{-1}\mathbf{G}(\theta_n)\mathbf{M}^{-\top}$.

3. Update using the estimated θ_n:

$$\mathbf{C}_{n|n-1} = \hat{\mathbf{C}}_{n|n-1} + \hat{\mathbf{G}}(\theta_n).$$

4. Condition:

$$\mathbf{m}_{n|n} = \mathbf{m}_{n|n-1} + \mathbf{C}_{n|n-1}\mathbf{H}_n^\top \left(\mathbf{H}_n \mathbf{C}_{n|n-1}\mathbf{H}_n^\top + \sigma_n^2 \mathbf{I}\right)^{-1}(\mathbf{y}_n - \mathbf{H}_n \mathbf{m}_{n|n-1}),$$

$$\mathbf{C}_{n|n} = \mathbf{C}_{n|n-1} - \mathbf{C}_{n|n-1}\mathbf{H}_n^\top \left(\mathbf{H}_n \mathbf{C}_{n|n-1}\mathbf{H}_n^\top + \sigma_n^2 \mathbf{I}\right)^{-1}\mathbf{H}_n \mathbf{C}_{n|n-1}.$$

   Thus $p(\mathbf{u}^n \mid \mathbf{y}_{1:n}, \theta_{1:n}, \sigma_{1:n}, \Lambda) = \mathcal{N}(\mathbf{m}_{n|n}, \mathbf{C}_{n|n})$.

3. Nonlinear, time-dependent statFEM

A general nonlinear PDE with stochastic forcing can be expressed as:

$$\begin{cases} \partial_t u + L_\Lambda u + F_\Lambda(u) + \xi_\theta = 0, & \xi \sim \mathcal{GP}(0, k_\theta(x, x') \cdot \delta(t - t')), \\ u := u(x, t), \quad x \in \Omega \subset \mathbb{R}^d, & t \in [0, T]. \end{cases} \tag{3}$$

The nonlinear statFEM construction is then as follows, assuming the separable covariance structure as in Eq. (2). As previous we start with the spatial discretization to give a semidiscrete problem (a vector SDE) and then proceed to the fully discrete solution via finite differences in time. We begin by multiplying by test functions $\psi \in V$ and integrating over the problem domain.

$$\langle \partial_t u, \psi \rangle + \langle L_\Lambda u, \psi \rangle + \langle F_\Lambda(u), \psi \rangle + \langle \xi_\theta, \psi \rangle = 0.$$

We next divide the domain Ω into finite elements on a given mesh and look for solutions in terms of a finite set of trial functions $\{\phi_i\}_{i \in I}$ against test functions $\{\psi_i\}_{i \in I}$ as before. We expand solutions in terms of these basis functions, $u_h(x, t) = \sum_{i \in I} u_{h,i}(t) \phi_i(x)$, to give the updated weak form:

$$\langle \partial_t u_h, \psi_j \rangle + \langle L_\Lambda u_h, \psi_j \rangle + \langle F_\Lambda(u_h), \psi_j \rangle + \langle \xi_\theta, \psi_j \rangle = 0,$$

which now defines a nonlinear, coupled system of stochastic differential equations. In general one can make no comment on the distributional form of the resultant probability measure on function space due to the nonlinear F_Λ. Following the derivations given in the linear case above we can write this system as a nonlinear vector SDE:

$$\mathbf{M} \, \mathrm{d}\mathbf{u} + \mathcal{F}_\Lambda(\mathbf{u}) \mathrm{d}t + \mathrm{d}\boldsymbol{\beta}_t = 0,$$

where $\mathbf{M}$ is the mass matrix and $\mathcal{F}_\Lambda$ is some nonlinear vector function that encodes the action of L_Λ and F_Λ.

Now, we discretize in time using Euler methods. The explicit Euler method at $\mathbf{u}^n := \mathbf{u}(n\Delta_t)$ gives

$$\mathbf{M} \left(\mathbf{u}^n - \mathbf{u}^{n-1} \right) + \Delta_t \mathcal{F}_\Lambda(\mathbf{u}^{n-1}) + \mathbf{e}_{n-1} = 0,$$

in which $\mathbf{e}_{n-1} = \boldsymbol{\beta}_n - \boldsymbol{\beta}_{n-1} \sim \mathcal{N}(0, \Delta_t \mathbf{G}(\theta))$ are i.i.d. Gaussian (timesteps are equal through the simulation). We can also use the implicit Euler

$$\mathbf{M} \left(\mathbf{u}^n - \mathbf{u}^{n-1} \right) + \Delta_t \mathcal{F}_\Lambda(\mathbf{u}^n) + \mathbf{e}_{n-1} = 0,$$

or the Crank-Nicolson

$$\mathbf{M} \left(\mathbf{u}^n - \mathbf{u}^{n-1} \right) + \Delta_t \mathcal{F}_\Lambda(\mathbf{u}^{n+1/2}) + \mathbf{e}_{n-1} = 0,$$

with $\mathbf{u}^{n+1/2} := (\mathbf{u}^{n+1} + \mathbf{u}^n)/2$, if stability of the time integration is needed. We can represent the above as some nonlinear updating equation:

$$\mathcal{F}_\Lambda(\mathbf{u}^n, \mathbf{u}^{n-1}) + \mathbf{e}_{n-1} = 0,$$

recycling the notation for the nonlinear vector-valued function $\mathcal{F}_\Lambda$. This system of equations requires solving at each timestep (via linear solvers or nonlinear Newton/quasi-Newton methods) and the solution will define the prior over the FEM coefficients at each time $n\Delta_t$. Obviously there is much development required to go from the weak form to having a timestepping regime in terms of the FEM coefficients, however for brevity we will just deal with the above (or variants thereof). Statisticians will note that from here on in we are essentially studying a high-dimensional nonlinear state-space model. We now turn to describing the data assimilation procedure.

4. Computing the posterior

Now, having the prior, we wish to condition on data to yield the posterior $p(\mathbf{u}^n \mid \mathbf{y}_{1:n}, \theta_{1:n}, \sigma_{1:n}, \Lambda)$, describing the belief in the FEM solution conditioned on data observed up to and including time $n\Delta_t$. We begin by stipulating the data generating process as, at each n, $\mathbf{y}_n = \mathbf{H}_n \mathbf{u}^n + \boldsymbol{\eta}_n$ in which

- $\mathbf{y}_n \in \mathbb{R}^N$ is the observed data at time $n\Delta_t$.
- $\mathbf{H}_n : \mathbb{R}^M \to \mathbb{R}^N$ is the observation operator that maps from the FEM solution mesh to the observed points $\mathbf{x}_{\text{obs}}$.
- $\mathbf{u}^n \in \mathbb{R}^M$ is the FEM coefficients at time n.
- $\boldsymbol{\eta}_n$ is a noise process that represents the measurement error for the observed values. This is a Gaussian $\boldsymbol{\eta}_n \sim \mathcal{N}(\mathbf{0}, \sigma_n^2 \mathbf{I})$ and thus so is the likelihood $p(\mathbf{y}_n \mid \mathbf{u}^n, \sigma_n, \Lambda) = \mathcal{N}(\mathbf{H}_n \mathbf{u}^n, \sigma_n^2 \mathbf{I})$.

A. Extended Kalman filter (EKF). At time n we start with the previous filtering distribution

$$p(\mathbf{u}^{n-1} \mid \mathbf{y}_{1:n-1}, \theta_{1:n-1}, \sigma_{1:n-1}, \Lambda) \sim \mathcal{N}(\mathbf{m}_{n-1|n-1}, \mathbf{C}_{n-1|n-1}),$$

then proceed as follows

1. Compute the prediction step for the mean by solving

$$\mathcal{F}_\Lambda(\mathbf{m}_{n|n-1}, \mathbf{m}_{n-1|n-1}) = \mathbf{0},$$

for $\mathbf{m}_{n|n-1}$, and computing the so-called forward covariance:

$$\hat{\mathbf{C}}_{n|n-1} = (\mathbf{J}^n)^{-1}\left(\mathbf{J}^{n-1}\mathbf{C}_{n-1|n-1}(\mathbf{J}^{n-1})^\top\right)(\mathbf{J}^n)^{-\top},$$

The Jacobians $\mathbf{J}^n = \frac{\partial}{\partial \mathbf{u}^n}\mathcal{F}_\Lambda(\mathbf{u}^n, \mathbf{u}^{n-1})$, $\mathbf{J}^{n-1} = \frac{\partial}{\partial \mathbf{u}^{n-1}}\mathcal{F}_\Lambda(\mathbf{u}^n, \mathbf{u}^{n-1})$ are evaluated at the solutions $\mathbf{m}_{n|n-1}$, $\mathbf{m}_{n-1|n-1}$. The reason we do this "half" prediction step is that the parameters θ_n are as yet unknown and must be estimated. The full prediction covariance is written as:

$$\mathbf{C}_{n|n-1} = \hat{\mathbf{C}}_{n|n-1} + \Delta_t(\mathbf{J}^n)^{-1}\mathbf{G}(\theta_n)(\mathbf{J}^n)^{-\top}.$$

2. Maximize the log-marginal posterior

$$\max_{\theta_n, \sigma_n}\{\log p(\mathbf{y}_n \mid \mathbf{y}_{1:n-1}, \theta_{1:n}, \sigma_{1:n}, \Lambda) + \log p(\theta_n) + \log p(\sigma_n)\}$$

to obtain estimated parameters θ_n, σ_n. The marginal likelihood can be written out as:

$$p(\mathbf{y}_n \mid \mathbf{y}_{1:n-1}, \theta_{1:n}, \sigma_{1:n}, \Lambda) = \mathcal{N}\left(\mathbf{H}_n\mathbf{m}_{n|n-1}, \mathbf{H}_n\hat{\mathbf{C}}_{n|n-1}\mathbf{H}_n^\top + \Delta_t\mathbf{H}_n\hat{\mathbf{G}}(\theta_n)\mathbf{H}_n^\top + \sigma_n^2\mathbf{I}\right)$$

where

$$\hat{\mathbf{G}}(\theta_n) = (\mathbf{J}^n)^{-1}\mathbf{G}(\theta_n)(\mathbf{J}^n)^{-\top}.$$

In this form we can calculate derivatives of marginal likelihood covariance — using well-known formulae from Gaussian process regression (see (3), Chapter 5).

3. Using the estimated θ_n, compute the full prediction covariance $\mathbf{C}_{n|n-1}$:

$$\mathbf{C}_{n|n-1} = \hat{\mathbf{C}}_{n|n-1} + \Delta_t\hat{\mathbf{G}}(\theta_n).$$

4. Complete a standard Kalman update, conditioning on $\mathbf{y}_n$:

$$\begin{aligned} p(\mathbf{u}^n \mid \mathbf{y}_{1:n}, \theta_{1:n}, \sigma_{1:n}, \Lambda) &\propto p(\mathbf{y}_n \mid \mathbf{u}^n, \sigma_n)p(\mathbf{u}^n \mid \mathbf{y}_{1:n-1}, \theta_{1:n}, \Lambda) \\ &= \mathcal{N}(\mathbf{m}_{n|n}, \mathbf{C}_{n|n}). \end{aligned}$$

where

$$\begin{aligned} \mathbf{m}_{n|n} &= \mathbf{m}_{n|n-1} + \mathbf{C}_{n|n-1}\mathbf{H}_n^\top\left(\mathbf{H}_n\mathbf{C}_{n|n-1}\mathbf{H}_n^\top + \sigma_n^2\mathbf{I}\right)^{-1}(\mathbf{y}_n - \mathbf{H}_n\mathbf{m}_{n|n-1}), \\ \mathbf{C}_{n|n} &= \mathbf{C}_{n|n-1} - \mathbf{C}_{n|n-1}\mathbf{H}_n^\top\left(\mathbf{H}_n\mathbf{C}_{n|n-1}\mathbf{H}_n^\top + \sigma_n^2\mathbf{I}\right)^{-1}\mathbf{H}_n\mathbf{C}_{n|n-1}. \end{aligned}$$

B. Ensemble Kalman filter (EnKF). At time n we start with the previous filtering distribution, which with N_{ens} ensemble members, is described by:

$$\left\{\mathbf{u}^{n-1,[i]}\right\}_i \sim p(\mathbf{u}^{n-1} \mid \mathbf{y}_{1:n-1}, \theta_{1:n-1}, \sigma_{1:n-1}, \Lambda).$$

Then proceed as follows

1. Compute the prediction step (without simulating stochastic forcing) for each $i = 1, \ldots, N_{ens}$

$$\mathcal{F}_\Lambda(\mathbf{u}^{n,[i]}, \mathbf{u}^{n-1,[i]}) = \mathbf{0},$$

and computing the so-called forward covariance:

$$\hat{\mathbf{C}}_{n|n-1} = \frac{1}{N_{ens}-1}\sum_{i=1}^{N_{ens}}\left(\mathbf{u}^{n,[i]} - \mathbf{m}_{n|n-1}\right)\left(\mathbf{u}^{n,[i]} - \mathbf{m}_{n|n-1}\right)^\top$$

where $\mathbf{m}_{n|n-1} = \frac{1}{N_{ens}}\sum_{i=1}^{N_{ens}}\mathbf{u}^{n,[i]}$. The reason we do this "half" prediction step is that the parameters θ_n are as yet unknown and must be estimated. The full prediction covariance is approximated as

$$\mathbf{C}_{n|n-1} = \hat{\mathbf{C}}_{n|n-1} + \Delta_t(\mathbf{J}^n)^{-1}\mathbf{G}(\theta_n)(\mathbf{J}^n)^{-\top},$$

where we employ this approximation in order to use analytical gradients in the optimization step.

2. Maximize the log-marginal posterior

$$\max_{\theta_n,\sigma_n} \{\log p(\mathbf{y}_n \mid \mathbf{y}_{1:n-1}, \theta_{1:n}, \sigma_{1:n}, \Lambda) + \log p(\theta_n) + \log p(\sigma_n)\}$$

to obtain estimated parameters θ_n, σ_n. The marginal likelihood can be written out as:

$$p(\mathbf{y}_n \mid \mathbf{y}_{1:n-1}, \theta_{1:n}, \sigma_{1:n}, \Lambda) = \mathcal{N}\left(\mathbf{H}_n \mathbf{m}_{n|n-1}, \mathbf{H}_n \hat{\mathbf{C}}_{n|n-1} \mathbf{H}_n^\top + \Delta_t \mathbf{H}_n \hat{\mathbf{G}}(\theta_n) \mathbf{H}_n^\top + \sigma_n^2 \mathbf{I}\right)$$

where $\hat{\mathbf{G}}(\theta_n) = (\mathbf{J}^n)^{-1} \mathbf{G}(\theta_n) (\mathbf{J}^n)^{-\top}$.

3. Using θ_n, compute the full prediction step:

$$\mathcal{F}_\Lambda(\mathbf{u}^{n,[i]}, \mathbf{u}^{n-1,[i]}) + \mathbf{e}_{n-1}^{[i]} = \mathbf{0},$$

for the predictions $\left\{\mathbf{u}^{n,[i]}\right\}_i$.

4. Then update the ensemble members by a Kalman shift

$$\mathbf{u}^{n,[i]} = \mathbf{u}^{n,[i]} + \mathbf{C}_{n|n-1} \mathbf{H}_n^\top \left(\mathbf{H}_n \mathbf{C}_{n|n-1} \mathbf{H}_n^\top + \sigma_y^2 \mathbf{I}\right)^{-1} \left(\mathbf{y}_n + \boldsymbol{\eta}_n^{[i]} - \mathbf{H}_n \mathbf{u}^{n,[i]}\right),$$

$$\mathbf{C}_{n|n-1} = \frac{1}{N_{ens} - 1} \sum_{i=1}^{N_{ens}} \left(\mathbf{u}^{n,[i]} - \mathbf{m}_{n|n-1}\right) \left(\mathbf{u}^{n,[i]} - \mathbf{m}_{n|n-1}\right)^\top, \quad \mathbf{m}_{n|n-1} = \frac{1}{N_{ens}} \sum_{i=1}^{N_{ens}} \mathbf{u}^{n,[i]},$$

to give the posterior ensemble $\left\{\mathbf{u}^{n,[i]}\right\}_i \sim p(\mathbf{u}^n \mid \mathbf{y}_{1:n}, \theta_n, \sigma_n, \Lambda)$.

C. Discussion of the method. Discretization of the covariance $\mathbf{G}(\theta)$ requires some care to implement as this is a $2d$ dimensional integral and in general does not give a sparse matrix as C_θ is a positive definite integral operator on Hilbert space. For large problems it may be necessary to impose some sort of sparsity constraint. This can be done e.g. by assuming space-time white noise to give

$$\begin{aligned}\mathbf{G}(\theta)_{ij} = \langle \psi_i, C_\theta \psi_j \rangle &= \int_\Omega \psi_i(x) \int_\Omega \delta(x, x') \psi_j(x') \, \mathrm{d}x' \, \mathrm{d}x \\ &= \int_\Omega \psi_i(x) \psi_j(x) \, \mathrm{d}x = \mathbf{M}_{ij},\end{aligned}$$

so the mass matrix $\mathbf{M}$ is the covariance matrix. Localization (see, e.g., (7)) may also be used to enforce a sparsity constraint and remove spurious correlations that may arise from small ensemble sizes (if the ensemble method is used). Brute force setting entries to zero, below some threshold value, could also be used.

Furthermore often products of the form $\mathbf{A}^{-1}\mathbf{G}(\theta)\mathbf{A}^{-\top}$ must be formed. If taking a truncated eigendecomposition of $\mathbf{G}(\theta)$ is not too problematic (using e.g. Lanczos iterations (8)) then one can approximate

$$\mathbf{A}^{-1}\mathbf{G}(\theta)\mathbf{A}^{-\top} \approx \mathbf{A}^{-1}\mathbf{Q}\mathbf{D}\mathbf{Q}^\top\mathbf{A}^{-\top} = (\mathbf{A}^{-1}\mathbf{Q})\mathbf{D}(\mathbf{A}^{-1}\mathbf{Q})^\top,$$

meaning many of matrix solves of $\mathbf{A}$ can be avoided by taking this low-rank approximation. This may be especially beneficial when $\mathbf{G}(\theta)$ has some additional structure than can be made use of to quickly compute its eigendecomposition (e.g. sparsity or Toeplitz structure).

The parameters θ_n and σ_n require estimation at each time n and are assumed to be independent across time. They are estimated by maximizing the log-marginal posterior (3):*

$$\log p(\theta_n, \sigma_n \mid \mathbf{y}_{1:n}, \theta_{1:n-1}, \sigma_{1:n-1}) \propto \log p(\mathbf{y}_n | \mathbf{y}_{1:n-1}, \theta_{1:n}, \sigma_{1:n}) + \log p(\theta_n) + \log p(\sigma_n)$$

where the marginal likelihood at time n is approximated with the EKF approximation

$$p(\mathbf{y}_n \mid \mathbf{y}_{1:n-1}, \theta_n, \sigma_n, \Lambda) = \mathcal{N}\left(\mathbf{H}_n \mathbf{m}_{n|n-1}, \mathbf{H}_n \hat{\mathbf{C}}_{n|n-1} \mathbf{H}_n^\top + \Delta_t \mathbf{H}_n \hat{\mathbf{G}}(\theta_n) \mathbf{H}_n^\top + \sigma_n^2 \mathbf{I}\right).$$

Unless otherwise mentioned we use the priors

$$\tau_n \sim \mathcal{N}_+(1, 1^2), \quad \ell_n \sim \mathcal{N}_+(1, 1^2), \quad \sigma_n \sim \mathcal{N}_+(0, 1^2). \qquad [4]$$

We choose to introduce prior information in order to regularize the optimization problem. Anecdotally, appropriate choices of these priors makes the optimization much better-behaved.

If parameters are assumed to be constant across time then the updating procedures provided above could be modified to account for this. One possibility is outlined in (6) and is based on optimizing the full likelihood:

$$\log p(\mathbf{y}_{1:N} \mid \theta, \sigma) = -\frac{1}{2} \sum_{n=1}^N \log |\boldsymbol{\Sigma}_n(\theta, \sigma)| - \frac{1}{2} \sum_{n=1}^N \left(\mathbf{y}_n - \mathbf{H}_n \mathbf{m}_{n|n-1}\right)^\top \boldsymbol{\Sigma}_n(\theta, \sigma)^{-1} \left(\mathbf{y}_n - \mathbf{H}_n \mathbf{m}_{n|n-1}\right),$$

in which $\boldsymbol{\Sigma}_n(\theta, \sigma) = \mathbf{H}_n \hat{\mathbf{C}}_{n|n-1} \mathbf{H}_n^\top + \Delta_t \mathbf{H}_n \hat{\mathbf{G}}(\theta) \mathbf{H}_n^\top + \sigma^2 \mathbf{I}$. One could use the above filtering algorithms with fixed parameters to evaluate this likelihood, also analytically calculating gradients on the way through. Priors can also be incorporated. Optimization can then proceed via standard methods (e.g. L-BFGS-B as we use for the marginal likelihood) noting that each likelihood (and gradient) evaluation requires computing the filtered distribution $p(\mathbf{u}^N \mid \mathbf{y}_{1:N}, \theta, \sigma)$ for fixed parameters θ and σ.

*Using Gaussian priors gives the Tikhonov regularization of the marginal likelihood. Other priors are also commonly used, see e.g. Chapter 10 of (9)

5. statFEM for KdV

We now illustrate the above using the eKdV equation, given by

$$\begin{cases} u_t + \alpha u u_x + \beta u_{xxx} + c u_x + \nu u + \xi_\theta = 0, \\ u := u(x,t), \quad x \in [0,L], \quad t \in [0,T], \\ u(x,t) = u(x+L,t), \\ u(x,0) := u_0(x). \end{cases}$$

For the deterministic problem, we discretize using the scheme outlined in (10). Start by defining the time grid as n_t evenly spaced points $(0, \Delta_t, 2\Delta_t, \ldots, (n_t-1)\Delta_t)^\top$, with $u^n(x) = u(x, n\Delta_t)$. We use Crank-Nicolson for time integration and continue as usual by multiplying with test functions $\psi \in V^0$ (V^0 some appropriate function space) and integrating with respect to x over $[0,L]$ to give the weak form

$$\langle u^{n+1} - u^n, \psi\rangle + \Delta_t \alpha \langle u^{n+1/2} u_x^{n+1/2}, \psi\rangle + \Delta_t \beta \langle u_{xxx}^{n+1/2}, \psi\rangle + \Delta_t c \langle u_x^{n+1/2}, \psi\rangle + \Delta_t \nu \langle u^{n+1/2}, \psi\rangle + \langle \xi_\theta^n - \xi_\theta^{n-1}, \psi\rangle = 0.$$

Where $\xi_\theta^n - \xi_\theta^{n-1} \sim \mathcal{GP}(0, \Delta_t k_\theta(x,x'))$ with k a squared-exponential kernel. To minimize the order of derivatives split the above into a system of three first order equations:

$$\begin{gathered} \langle u^{n+1} - u^n, \psi\rangle + \Delta_t \alpha \langle u^{n+1/2} u_x^{n+1/2}, \psi\rangle + \Delta_t \beta \langle w_x^{n+1/2}, \psi\rangle + \Delta_t c \langle u_x^{n+1/2}, \psi\rangle + \Delta_t \nu \langle u^{n+1/2}, \psi\rangle + \langle \xi_\theta^n - \xi_\theta^{n-1}, \psi\rangle = 0, \\ \langle u_x^{n+1/2}, \psi\rangle = \langle v^{n+1/2}, \psi\rangle, \\ \langle v_x^{n+1/2}, \psi\rangle = \langle w^{n+1/2}, \psi\rangle. \end{gathered}$$

This is necessary as we use P_0 test functions $\{\psi_i\}_i$ and P_1 trial functions $\{\phi_i\}_i$. The solutions to the deterministic problem thus reside in the space $V_h^1 = \mathrm{span}\,(\{\phi_i\}_i) \subset C^1([0,L])$.

To solve the above system at each time requires the solution of a nonlinear system of equations which is implemented using Newton's method. Convergence is typically achieved in < 5 Newton iterations. It is noted in (10) that solving the system directly as opposed to linearizing about previous solutions avoids numerical dissipation present in other schemes. When using the statFEM for KdV, the covariance $\mathbf{G}(\theta)$ is evaluated with the trapezoidal rule which is known to be exponentially convergent for periodic functions (11).

A. FEM approximation convergence. To compute discretization error estimates for the EKF prior mean we make use of the fact that KdV is integrable with "nice" expressions of the exact solution $u(x,t)$ for judicious choice of coefficients and initial conditions. In this study we chose $\alpha = 1$, $\beta = 10^{-3}$, and initial conditions

$$u(x,0) = \frac{3}{2}\mathrm{sech}^2\left(\frac{1}{2}\sqrt{\frac{1}{2\beta}}(x-1)\right)$$

which gives the analytical solution (assuming that $u = 0$, $\partial_x u = 0$, as $x \to \pm\infty$)

$$u(x,t) = \frac{3}{2}\mathrm{sech}^2\left(\frac{1}{2}\sqrt{\frac{1}{2\beta}}\left(x - 1 - \frac{t}{2}\right)\right).$$

We solve on $x \in [0,3]$ with periodic boundary conditions; this mimics solving on the real line. We compute the L^2 error $\|u - u_h\|_2$ for FEM discretized solutions u_h using $200, 400, 600, 800, 1000$, and 2000 mesh nodes, after running 100 timesteps with $\Delta_t = 10^{-3}$. The resultant errors are shown in Figure S1a and give error $\mathcal{O}(h^2)$, as expected with P_1 elements.

B. Simulation study. We now present the consistency of the prior for the simulation study. In this case we take the same settings as in the main text: $\alpha = 1$, $\beta = 0.01$, $c = 0$, and investigate the effect of reducing the ξ_θ scale parameter $\tau_n \equiv \tau$ for equal $\ell_n \equiv \ell$. These parameters are assumed constant for all n. Results are shown in Figure S1b, computed using the EnKF method, and demonstrate that (a) uncertainty grows as the simulation carries on; and (b) the reduction of the scale parameter τ results in the uncertainty bands contracting about the FEM solution. The prior mean converges to the FEM solution as τ is decreased. However there is some damping for larger τ values. We posit that damping is due to the dispersive behaviour of KdV. By perturbing solutions we cause the ensemble members to drift apart as the simulation runs.

C. Experimental data. We set the noise variance after Fourier filtering the experimental data at the first wave-gauge, attenuating to the first 100 frequencies only. Estimation of this variance gives the time-averaged constant variance $\sigma_n^2 \approx 1.3588 \times 10^{-8}$. In order to estimate the other hyperparameters we use a projection method to address potential small data problems. At each timestep, we have three measurements in space, which is less than ideal when trying to learn hyperparameters. To inflate the dataset we project forward using a least-squares projection:

$$y_i^n = a_n + b_n \mathbf{u}^n(x_i),$$

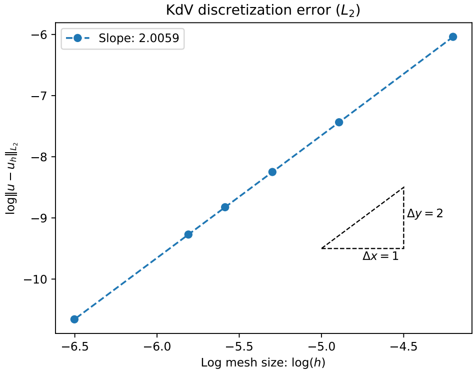

(a) KdV L^2 error estimates.

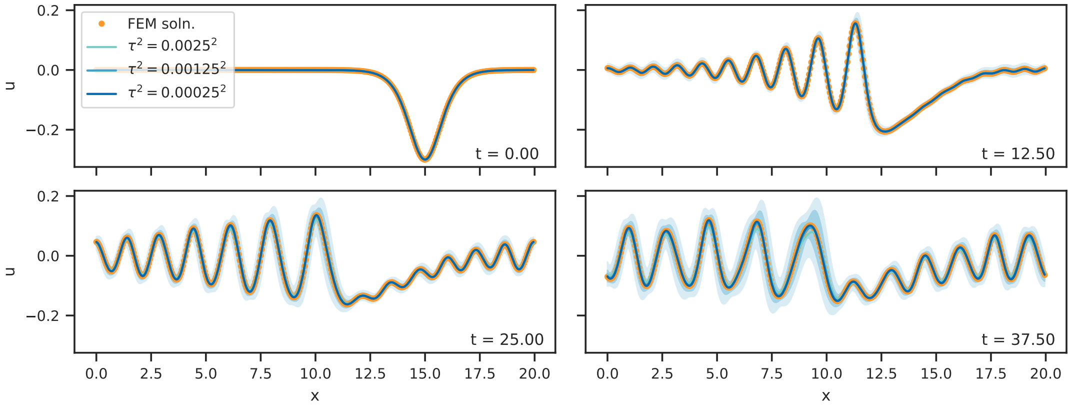

(b) Cubic KdV prior consistency plots.

which requires estimating the a_n, b_n at each iteration. This gives the best least-squares linear projection from the current prediction to the data, to give us a predicted dataset $\tilde{\mathbf{y}}_n$ via using the linear shift:

$$\tilde{\mathbf{y}}_n = a_n + b_n \mathbf{u}^n.$$

Using this method we can extend our dataset to now be as large as our FEM solution grid. In the paper we project to a grid of 100 points, uniformly spaced across the solution grid. This is only for the parameter estimation step. We do not use these values as the data in the analysis/conditioning step of the Kalman filter; we just use this to estimate the parameters, to improve the conditioning of the problem. We use the same weakly informative priors

$$\tau_n \sim \mathcal{N}_+(1, 1^2), \quad \ell_n \sim \mathcal{N}_+(1, 1^2).$$

6. Further examples

In this section we demonstrate the application of the method on a set of PDEs. We use two well-known systems: the Kuramoto-Sivashinsky equation and the 2D Burgers' equation. The general theme of this section is that we generate some mismatched data and then condition on it after jittering it with synthetic Gaussian observational error. Different to KdV, we discretize all equations with `Fenics` (12) and use the EKF in both examples. Scripts to run these examples are included in the accompanying GitHub repository, available at https://github.com/connor-duffin/statkdv-paper.

A. Kuramoto-Sivashinsky. The Kuramoto-Sivashinsky (KS) equation is a chaotic, biharmonic PDE that is used to model pattern formation in a variety of physical contexts (13). In this paper we consider the 1D KS equation, which is given by:

$$\begin{cases} u_t + uu_x + \nu u_{xx} + u_{xxxx} = 0, \\ u := u(x,t), \ x \in [0, 32\pi], \ t \in [0, 100], \\ u(0,t) = u(32\pi, t). \end{cases} \tag{5}$$

We discretize using P_1 trial and test functions with 512 elements in space, and an implicit Euler timestepping scheme ($\Delta_t = 0.02$). To deal with the fourth order system we split into a system of coupled PDEs (similar to KdV), to give the semi-discrete

(time-discretized) equations

$$\langle u^n - u^{n-1}, v\rangle + \Delta_t \langle u^n u_x^n, v\rangle - \Delta_t \nu \langle u_x^n, v_x\rangle - \Delta_t \langle w_x^n, v_x\rangle = 0,$$
$$-\langle u_x^n, v_x\rangle - \langle w^n, v\rangle = 0,$$

for test functions $v \in V$.

We generate data with an under-damped model, with $\nu = 0.95$. StatFEM conditions on 52 observations per timestep, which have simulated Gaussian error, $\boldsymbol{\eta}_n \sim \mathcal{N}(0, 0.05^2)$. For the statFEM model, we assume the standard base model

$$u_t + uu_x + u_{xx} + u_{xxxx} + \xi_\theta = 0, \ \xi_\theta \sim \mathcal{GP}(0, C_\theta),$$

in which mismatch is induced by different dissipation magnitudes (0.95 to 1). As before, set the covariance of ξ_θ to $\mathbb{E}\left[\xi_\theta(x,t)\xi_\theta(x',t')\right] = k_\theta(x,x') \cdot \delta(t,t')$, with k_θ given by a squared exponential covariance function now with parameters $\theta_n = (\tau_n, \ell_n)$ and σ_n estimated at each timestep n. The data generating process is given by $\mathbf{y}_n = \mathbf{H}\mathbf{u}^n + \boldsymbol{\eta}_n$; the data are generated according to the KS model and a measurement error. The initial conditions are set from running the data-generating process (i.e. Eq. (5) for $\nu = 0.95$), initialized with $u(x,0) = \sin(x/16)$, for 2000 timesteps to skip over transient behaviour. In this case to compute the covariance matrix $\mathbf{G}(\theta_n)$ we approximate

$$\begin{aligned}
\mathbf{G}(\theta_n)_{ij} &= \int_\Omega \psi_i(x) \int_\Omega k_\theta(x,x')\psi_j(x')\,\mathrm{d}x'\,\mathrm{d}x \\
&\approx \sum_m \int_\Omega \psi_i(x) \sum_n \int_\Omega \psi_m(x) k_\theta(x_m, x_n)\psi_n(x')\psi_j(x')\,\mathrm{d}x'\,\mathrm{d}x \\
&\approx \sum_m \int_\Omega \psi_i(x)\psi_m(x) \sum_n \int_\Omega k_\theta(x_m, x_n)\psi_n(x')\psi_j(x')\,\mathrm{d}x'\,\mathrm{d}x \\
&= \sum_m \mathbf{M}_{im} \sum_n \mathbf{K}(\theta_n)_{mn}\mathbf{M}_{nj},
\end{aligned}$$

so $\mathbf{G}(\theta_n) = \mathbf{M}\mathbf{K}(\theta_n)\mathbf{M}^\top$ for mass matrix $\mathbf{M}$ and covariance matrix $\mathbf{K}(\theta_n)_{ij} = k_\theta(x_i, x_j)$ for FEM node locations x_i, x_j.

First, we analyse the discretization error estimates for the EKF prior mean. In this case KS is non-integrable (14), so we use a refined FEM solution with 4096 mesh nodes as the reference solution, and compute the L^2 error estimates $\|u - u_h\|_2$ for FEM solutions u_h with $\{32, 64, 128, 256, 512\}$ mesh nodes. We compute 100 timesteps with $\Delta_t = 10^{-3}$ and then evaluate the L^2 error norm in each case. The results are shown in Figure S2a and show error $\mathcal{O}(h^2)$ as with KdV.

To compute the filtering distribution $p(\mathbf{u}^n \mid \mathbf{y}_{1:n}, \boldsymbol{\theta}_{1:n}, \sigma_{1:n}, \Lambda)$ we use a modified version of the EKF algorithm, with the priors given in Eq. (4). This modification makes use of the eigendecomposition of the covariance matrices $\mathbf{C}_{n|n}$ and $\mathbf{G}(\theta_n)$ and is called the *low rank EKF* in (15). For the cost of a truncated eigendecomposition this avoids inverting the Jacobian many times. We use this approximation when computing the prediction covariances $\hat{\mathbf{C}}_{n|n-1}$ and $\hat{\mathbf{G}}(\theta_n)$ which are $(\mathbf{J}^n)^{-1}\mathbf{J}^{n-1}\mathbf{C}_{n|n-1}(\mathbf{J}^{n-1})^\top(\mathbf{J}^n)^{-\top}$ and $(\mathbf{J}^n)^{-1}\mathbf{G}(\theta_n)(\mathbf{J}^n)^{-\top}$ respectively. Taking an eigendecomposition results in products of the form

$$(\mathbf{J}^n)^{-1}\mathbf{Q}\boldsymbol{\Lambda}\mathbf{Q}^\top(\mathbf{J}^n)^{-\top} = (\mathbf{J}^n)^{-1}\mathbf{Q}\boldsymbol{\Lambda}\left((\mathbf{J}^n)^{-1}\mathbf{Q}\right)^\top$$

which, if the first k eigenvalue/vector pairs are computed, results in having to invert $\mathbf{J}^n$ k times. So if $k \ll M$ this can save some compute time. For this example we use the first 50 eigenvalue/vector pairs.

Results are shown in Figure S2. Figure S2b shows the posterior profiles for four times across the simulation. The initial conditions are the same and the posterior corrects for the difference between the data and the prior. The prior and posterior means, shown across the entire simulation grid in Figure S2c show completely different behaviour. This is likely due to KS being chaotic; small differences in the simulation early on are magnified by the end time. The estimated mismatch hyperparameters are shown in Figure S2d. The scale and length parameters each appear to reach stable estimates, which coincide with the prior mean. The noise (which has prior mean 0) is approximately identified at each iteration.

B. Burgers' equation. Burgers' equation is a nonlinear PDE that describes the balance of nonlinear steepening and viscous damping. The equation is often taken as a stepping-stone to the full simulation of the Navier-Stokes equations as it retains nonlinearity and viscous effects but is able to be solved in 1D (where the full Navier-Stokes requires at least 2D). In this subsection we study the 2D Burgers' equation given by

$$\begin{cases}
u_t + uu_x + vu_y = \frac{1}{\mathrm{Re}}\nabla^2 u, \\
v_t + uv_x + vv_y = \frac{1}{\mathrm{Re}}\nabla^2 v, \\
u := u(x,t), \ (x,y) \in [0,2]\times[0,2], \ t \in [0,5], \\
u(0,y,t) = u(2,y,t), \ u(x,0,t) = u(x,2,t), \\
v(0,y,t) = v(2,y,t), \ v(x,0,t) = v(x,2,t), \\
u(x,y,0) = v(x,y,0) = \sin(\pi(x+y)).
\end{cases} \qquad [6]$$

We discretize using P_1 trial and test functions and Crank-Nicolson in time. The semi-discretized version of Eq. (6) (discretized in time) is

$$\langle u^n - u^{n-1}, w_1 \rangle + \Delta_t \langle u^{n+1/2} u_x^{n+1/2}, w_1 \rangle + \Delta_t \langle v^{n+1/2} u_y^{n+1/2}, w_1 \rangle + \Delta_t \frac{1}{\mathrm{Re}} \langle \nabla u^{n+1/2}, \nabla w_1^{n+1/2} \rangle = 0$$
$$\langle v^n - v^{n-1}, w_2 \rangle + \Delta_t \langle u^{n+1/2} u_x^{n+1/2}, w_2 \rangle + \Delta_t \langle v^{n+1/2} u_y^{n+1/2}, w_2 \rangle + \Delta_t \frac{1}{\mathrm{Re}} \langle \nabla u^{n+1/2}, \nabla w_2^{n+1/2} \rangle = 0,$$

for testing functions w_1, w_2, with periodic boundary conditions.

Data $\mathbf{y}_n$ are generated by solving Eq. (6) with Reynolds number $\mathrm{Re} = 150$. The statFEM conditions on 102 observations per timestep, which are jittered with simulated observational error $\boldsymbol{\eta}_n \sim \mathcal{N}(0, 0.01^2)$. Data is observed on the velocity field u only, and the observation locations are shown on the mesh in Figure S3a. Note that in application more careful design of experiments may be necessary in order to choose these observation locations, especially for complex domains and/or more nonlinear regimes. Due to damping noise becomes more apparent as the simulation runs. The base model is taken to be Eq. (6) with the added unknown forcing on the evolution equation for u only

$$u_t + uu_x + vu_y = \frac{1}{\mathrm{Re}} \nabla^2 u + \xi_\theta,$$
$$v_t + uv_x + vv_y = \frac{1}{\mathrm{Re}} \nabla^2 v.$$

Mismatch is induced by setting $\mathrm{Re} = 100$ which in practice may occur because of discrepancy between measurements and modelling assumptions. The covariance of ξ_θ is given by $\mathbb{E}\left[\xi_\theta(x,t)\xi_\theta(x',t')\right] = k_\theta(x,x') \cdot \delta(t,t')$, with k_θ given by a squared exponential covariance function with parameters $\theta_n = (\tau_n, \ell_n)$. In this example we set $\ell_n = 1$ to avoid recomputing $\mathbf{G}(\theta_n)$ in each iteration, which is computed in the same way as in the KS example (i.e. using the mass matrix $\mathbf{G}(\theta_n) = \mathbf{M}\mathbf{K}(\theta_n)\mathbf{M}^\top$). The assumed data generating process is

$$\mathbf{y}_n = \mathbf{H} \begin{pmatrix} \mathbf{u}^n \\ \mathbf{v}^n \end{pmatrix} + \boldsymbol{\eta}_n \qquad [7]$$

and data are assumed to be generated according to the Burgers equation plus some measurement error.

In implementation we compute the posterior using a regular mesh with 64×64 elements in space (shown in Figure S3a) and timestep $\Delta_t = 0.01$. The L^2 discretization error for the initial conditions is shown in Figure S3b, estimated using gridsizes of $n_x = n_y \in \{16, 32, 64, 128, 256\}$ nodes (in each dimension). As with the previous examples we find that errors are $\mathcal{O}(h^2)$, where h is in this case the size of the individual elements. We use the initial condition as computing a highly refined solution becomes expensive due to the increasing number of mesh nodes in the solution. To compute the filtering distribution $p(\mathbf{u}^n \mid \mathbf{y}_{1:n}, \boldsymbol{\theta}_{1:n}, \sigma_{1:n}, \Lambda)$ we use the low-rank approximation as in the KS example, this time using the highest $k = 128$ eigenvector/eigenvalue pairs.

Results are shown in Figure S4. Note that in this section we only plot the u component, as this is the field on which the data is observed. The data generating process (Figure S4a) and posterior means (Figure S4b) show visual agreement, and the posterior variances (Figure S4c) indicate that uncertainty is greatest about the regions where the classical shocks develop (i.e. the regions of highest gradient in the solution surface) as well as the bottom right-hand corner which has the greatest distance from the nearest observation location. As the simulation runs these localized regions of highest uncertainty dissipate as a result of the viscous damping in the model due to the $\frac{1}{\mathrm{Re}}\nabla^2 u$ term.

The posterior surfaces shown in Figure S4d show the posterior means and the observed data points. The posterior mean shows agreement with the observed data. The normalized relative L^2 error $\|\bar{\mathbf{u}} - \mathbf{u}_{\mathrm{DGP}}\|_2 / \|\mathbf{u}_{\mathrm{DGP}}\|_2$ is also plotted in Figure S4e, for the prior mean and the posterior mean (this is approximated using FEM coefficients). The errors initially grow rapidly and then stabilise with time. Rapid initial increase in error is the same for the prior and posterior and after the filtering warm-up period — in which the noise is also overestimated (see Figure S4f) — the posterior error becomes lower than the prior and appears to increase at a lower rate. Increasing error is to be expected as we are not updating the model coefficients, only the numerical solution. Hence there will always be systematic differences between the data generating process and the posterior which conditioning on data can only partly account for. We conjecture this results in consistently increasing errors. Possible future work could investigate ways to estimate coefficients during the filtering procedure thus eliminating this source of misspecification.

Finally the parameter estimates are shown in Figure S4f. The true value of the noise is shown as a dashed orange band. After some warm-up time the parameters reach stable estimates with σ_n being slightly overestimated from the data. The estimates of the mismatch scale τ_n are more variable (similar to what is seen in the KdV examples) implying that the prediction step of the model varies in it's ability to replicate the data.

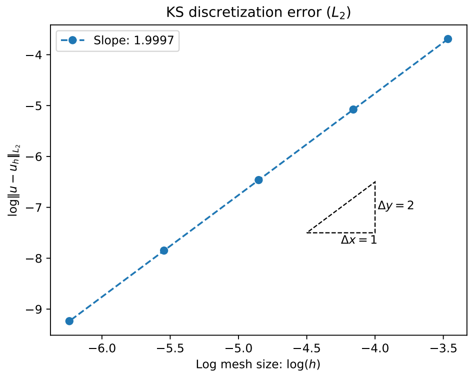

(a) KS L^2 error estimates.

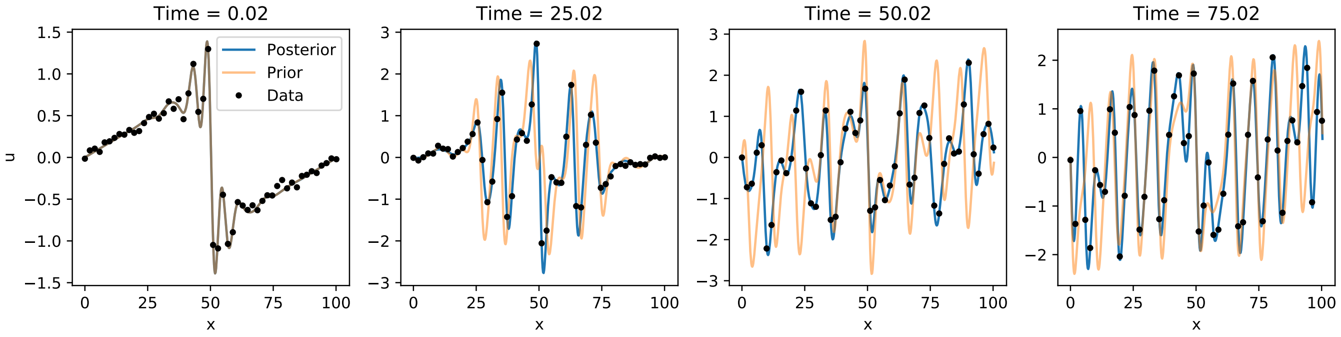

(b) Posterior mean and 95% credible intervals, prior mean, and observed data, for four evenly space times across the simulation.

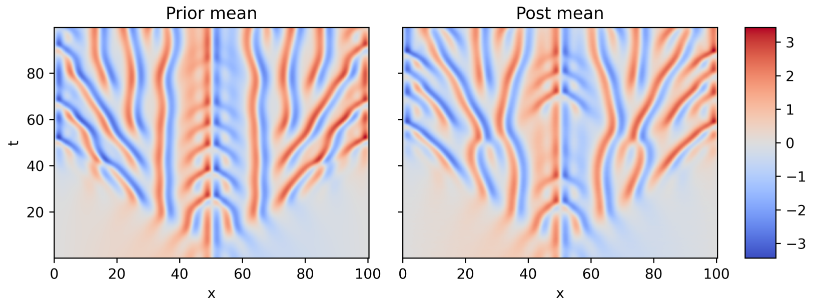

(c) Space-time view of prior and posterior means across the simulation grid. Conditioning on data gives a (visually) very different system

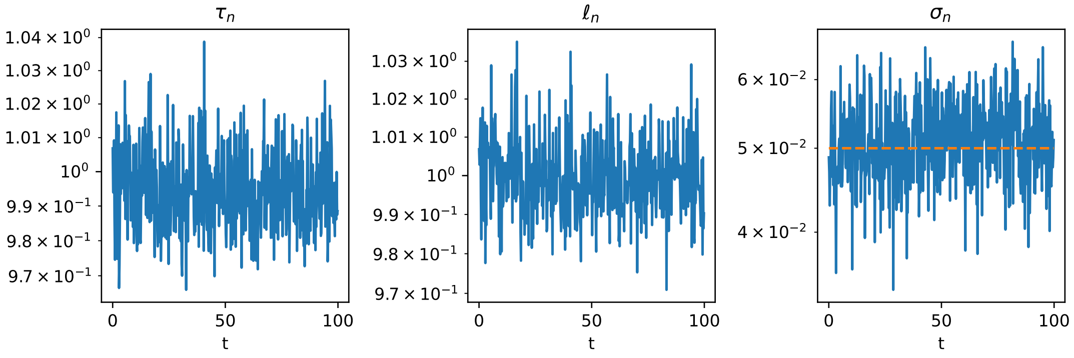

(d) Estimated covariance hyperparameters $(\tau_n, \ell_n, \sigma_n)$ for all times.

Fig. S2. KS equation posterior results.

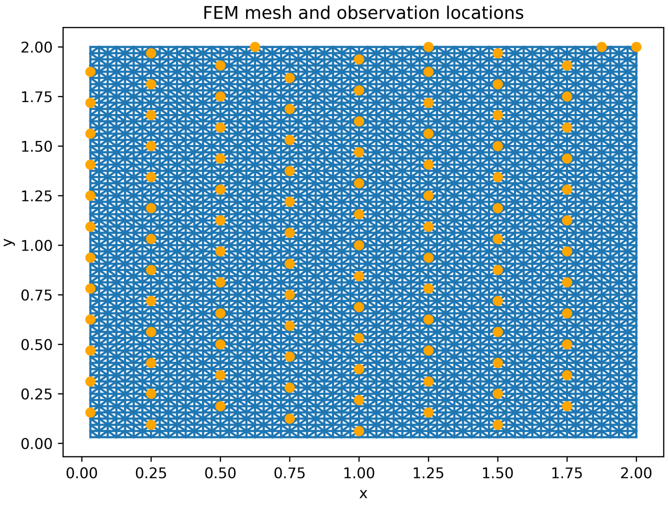

(a) Observation locations (orange) and FEM mesh (blue) for the Burgers example.

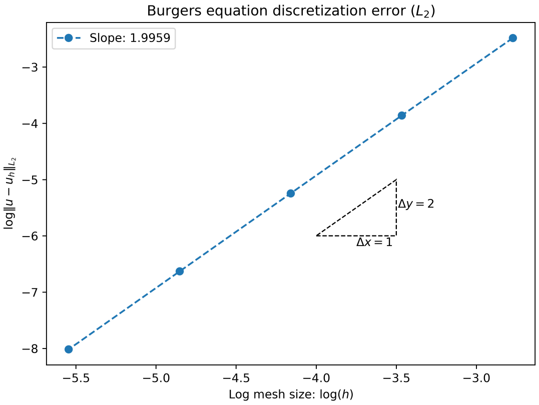

(b) L^2 convergence rate computed for the 2D Burgers equation (u component only).

Fig. S3. 2D Burgers simulation: settings and setup.

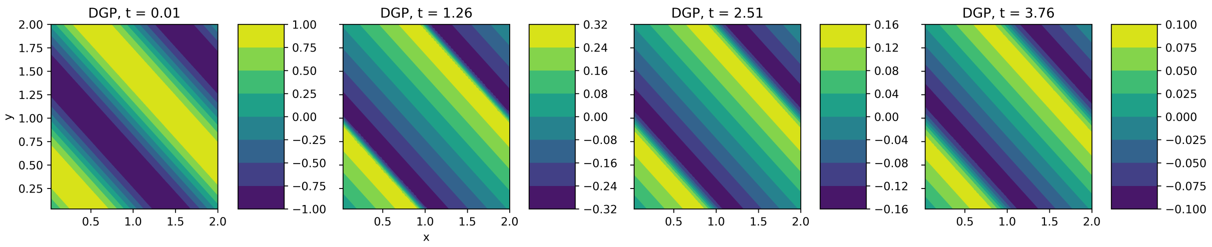

(a) Burgers DGP means for component u across all times.

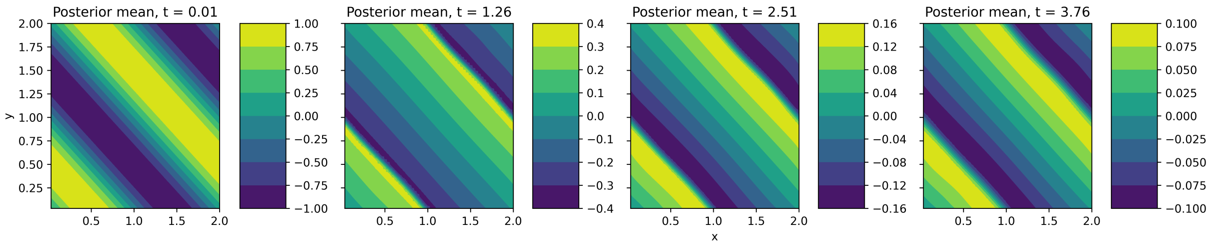

(b) Burgers posterior means for component u across all times.

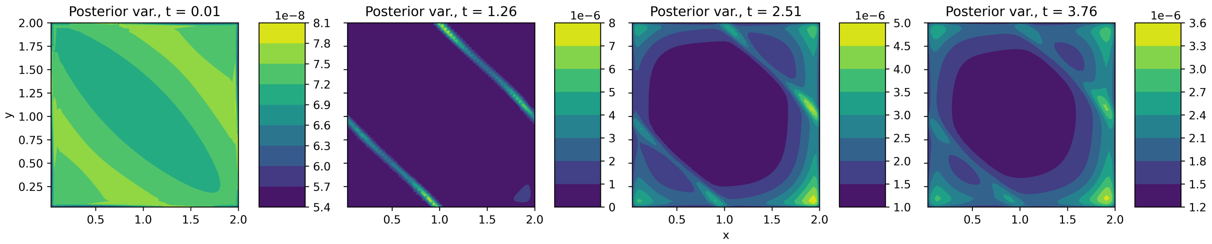

(c) Burgers posterior variances for component u on the mesh (using EKF).

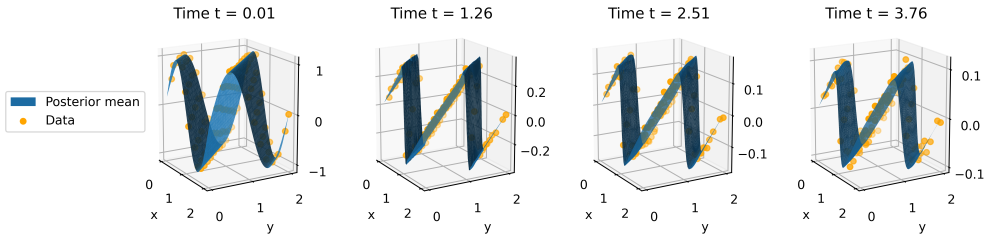

(d) Burgers equation surfaces for component u (shown as a blue surface) and observed data (orange points).

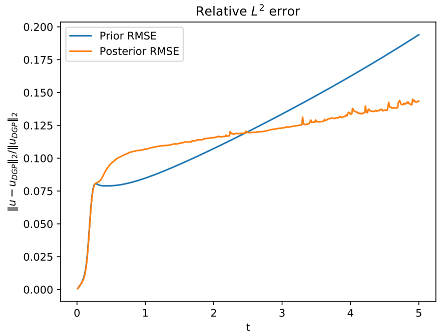

(e) Relative L^2 error between the prior/posterior mean and the known data generating process (on the u component only).

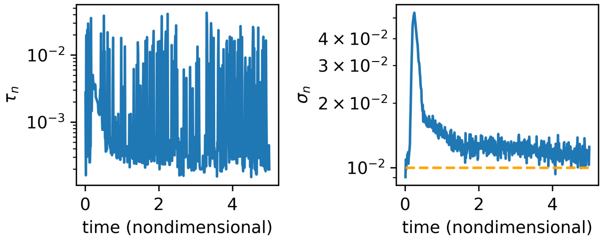

(f) Burgers parameter estimates. The dashed orange line shows the true value of the noise which appears slightly overestimated at each time. We conjecture this may be due to identifiability problems with the stochastic forcing magnitude.

Fig. S4. 2D Burgers equation results using the EKF.

References

1. Stuart AM (2010) Inverse problems: a Bayesian perspective. *Acta numerica* 19:451–559.
2. Evans LC (1998) *Partial Differential Equations.* (American Mathematical Society).
3. Williams CK, Rasmussen CE (2006) *Gaussian processes for machine learning.* (MIT press Cambridge, MA) Vol. 2.
4. Thomée V (2006) *Galerkin finite element methods for parabolic problems*, Springer series in computational mathematics. (Springer, Berlin ; New York) No. v. 25, 2nd ed edition.
5. Särkkä S, Solin A (2019) *Applied stochastic differential equations.* (Cambridge University Press) Vol. 10.
6. Shumway RH, Stoffer DS (2017) *Time Series Analysis and Its Applications: With R Examples*, Springer Texts in Statistics. (Springer International Publishing), 4 edition.
7. Houtekamer PL, Mitchell HL (2001) A Sequential Ensemble Kalman Filter for Atmospheric Data Assimilation. *Monthly Weather Review* 129(1):123–137.
8. Golub GH, Van Loan CF (1996) *Matrix Computations.* (The Johns Hopkins University Press), Third edition.
9. Stan Development Team (2020) *Stan User's Guide.*
10. Debussche A, Printems J (1999) Numerical simulation of the stochastic Korteweg–de Vries equation. *Physica D: Nonlinear Phenomena* 134(2):200–226.
11. Trefethen LN, Weideman J (2014) The exponentially convergent trapezoidal rule. *SIAM Review* 56(3):385–458.
12. Logg A, Mardal KA, Wells G (2012) *Automated solution of differential equations by the finite element method: The FEniCS book.* (Springer Science & Business Media) Vol. 84.
13. Hyman JM, Nicolaenko B (1986) The Kuramoto-Sivashinsky equation: A bridge between PDE's and dynamical systems. *Physica D: Nonlinear Phenomena* 18(1):113–126.
14. Conte R, Musette M (1989) Painleve analysis and Backlund transformation in the Kuramoto-Sivashinsky equation. *Journal of Physics A: Mathematical and General* 22(2):169–177.
15. Law KJH, Stuart AM (2012) Evaluating Data Assimilation Algorithms. *Monthly Weather Review* 140(11):3757–3782.
